# Chiral-at-Metal: Iridium(III) Tetrazole Complexes With Proton-Responsive P-OH Groups for CO_2_ Hydrogenation

**DOI:** 10.3389/fchem.2020.591353

**Published:** 2020-11-13

**Authors:** Edward Ocansey, James Darkwa, Banothile C. E. Makhubela

**Affiliations:** Research Centre for Synthesis and Catalysis, Department of Chemical Science, University of Johannesburg, Auckland Park, South Africa

**Keywords:** chiral-at-metal 1, tetrazole 2, iridium(III) complexes 3, CO_2_ Hydrogenation 4, NaHCO_3_ reduction, CO_2_ utilization, green chemistry

## Abstract

A rise in atmospheric CO_2_ levels, following years of burning fossil fuels, has brought about increase in global temperatures and climate change due to the greenhouse effect. As such, recent efforts in addressing this problem have been directed to the use of CO_2_ as a non-expensive and non-toxic single carbon, C_1_, source for making chemical products. Herein, we report on the use of tetrazolyl complexes as catalyst precursors for hydrogenation of CO_2_. Specifically, tetrazolyl compounds bearing P–S bonds have been synthesized with the view of using these as P^∧^N bidentate tetrazolyl ligands (**1**–**3**) that can coordinate to iridium(III), thereby forming heteroatomic five-member complexes. Interestingly, reacting the *P*,*N*′-bidentate tetrazolyl ligands with [Ir(_C_5_*Me*_5_)*Cl*_2_]2_ led to serendipitous isolation of chiral-at-metal iridium(III) half-sandwich complexes (**7**–**9**) instead. Complexes **7**–**9** were obtained via prior formation of non-chiral iridium(III) half-sandwich complexes (**4**–**6**). The complexes undergo prior P–S bond heterolysis of the precursor ligands, which then ultimately results in new half-sandwich iridium(III) complexes featuring monodentate phosphine co-ligands with proton-responsive P-OH groups. Conditions necessary to significantly affect the rate of P–S bond heterolysis in the precursor ligand and the subsequent coordination to iridium have been reported. The complexes served as catalyst precursors and exhibited activity in CO_2_ and bicarbonate hydrogenation in excellent catalytic activity, at low catalyst loadings (1 μmol or 0.07 mol% with respect to base), producing concentrated formate solutions (*ca* 180 mM) exclusively. Catalyst precursors with proton-responsive P-OH groups were found to influence catalytic activity when present as racemates, while ease of dissociation of the ligand from the iridium center was observed to influence activity in spite of the presence of electron-donating ligands. A test for homogeneity indicated that hydrogenation of CO_2_ proceeded by homogeneous means. Subsequently, the mechanism of the reaction by the iridium(III) catalyst precursors was studied using ^1^H NMR techniques. This revealed that a chiral-at-metal iridium hydride species generated *in situ* served as the active catalyst.

## Introduction

The utilization of fossil fuels as a source of energy has contributed toward human development (Saeidi et al., 2014). However, this dependence on fossil fuels has contributed to the gradual buildup of greenhouse gases such as CO_2_ in the atmosphere. This increase in the amount of greenhouse gases is a major contributor to the greenhouse effect that has caused increase in global temperatures and climate change (Huang and Tan, 2014; Saeidi et al., 2014). Increase in the amount of CO_2_ as a greenhouse gas in the atmosphere has necessitated the development of economical, safe, and efficient systems to capture and utilize it as a resource instead of disposing it as waste. Thus, CO_2_ serves as an attractive single carbon (C_1_) building block for making various compounds including formic acid, methanol, formamides, formaldehyde to name a few (Ma et al., 2009). Although CO_2_ is currently a C_1_ source in some industrial processes, like in the production of urea and salicylic acid, its thermodynamic stability still hinders its widespread utilization. Therefore, CO_2_ valorization requires electro- or photo-reduction or reacting it with high-energy substrates in the presence of catalysts. Catalytic hydrogenation of CO_2_ provides an avenue for CO_2_ fixation so as to unlock its full potential as a C_1_ source, thereby promoting its valorization (Jessop et al., 1995).

Various homogeneous and heterogeneous catalysts have been reported for the hydrogenation of CO_2_ to a variety of products. However, one of the best catalysts for the hydrogenation of CO_2_ is the trihydridoiridium P^∧^N^∧^P pincer complex, by Nozaki and co-workers (Tanaka et al., 2009), which served as an excellent catalyst. Despite the impressive catalytic activity shown by the Nozaki iridium catalyst (*ca* TONs as high as 3,500,000), it requires a strong base to achieve such high catalytic activity and productivity (200,000 h^−1^). Also, a strong base is used during the catalytic process, meaning isolation of the product (i.e., formic acid) requires use of a strong acid in order to convert the product formate to formic acid. Use of acids in chemical processes is not ideal as they are highly corrosive to equipment and they generate unwanted acid effluent. There is also the challenge of forming unwanted inorganic salts as by-products (Yasaka et al., 2010) Therefore, the development of highly active catalysts for the hydrogenation of carbon dioxide that use mild organic bases such as DBU is desirable (Makuve et al., 2019; Malaza and Makhubela, 2020).

Another class of catalysts for CO_2_ hydrogenation are iridium half-sandwich complexes. They have been shown to be effective catalysts for both CO_2_ hydrogenation and formic acid dehydrogenation (Leitner et al., 1994; Himeda et al., 2007, 2008; Boddien et al., 2011, 2012; Papp et al., 2011). In fact, Himeda and co-workers have demonstrated that the electron-donating ability of *N,N*′-bidentate ligands in half-sandwich iridium complexes enhances the catalytic activity of these complexes for the CO_2_ hydrogenation reaction through electronic effects (Himeda et al., 2007). More recently in 2017, Himeda and co-workers reported (cyclopentadienyl)iridium(III) complexes with pyridyl-diazole ligands that have hydroxyl electron-donating functional groups and an electron-rich diazole ring. The presence of the hydroxyl groups under basic conditions deprotonate to form strongly electron-donating oxyanions that activate dihydrogen through second sphere coordination effects, leading to the formation of the catalytically active iridium hydride in the CO_2_ hydrogenation reaction (Rakowski Dubois and Dubois, 2009; Wang et al., 2012; Suna et al., 2014). This is generally referred to as proton-responsive catalytic reactions. Both computational and experimentally observed studies have confirmed the participation of proton-responsive groups in the catalytic cycle of iridium hydride species in CO_2_ hydrogenation reactions (Suna et al., 2017). This, therefore, clearly indicates that the choice of ligand plays a vital role in the catalytic activity of complexes in CO_2_ hydrogenation reactions.

We postulated, at the beginning of this project, that iridium tetrazole complexes featuring NH groups are likely to display proton-responsive behavior and thus act as highly active CO_2_ hydrogenation catalysts. This formed the basis of our investigation and the current report (Ocansey et al., 2020). We are aware of several tetrazole MOFs, with possible proton-responsive NH groups, that have mostly been utilized in the synthesis of MOF for CO_2_ capture or CO_2_-assisted cycloaddition reactions, but none, to the best of our knowledge, has been used as CO_2_ hydrogenation catalyst (Pachfule and Banerjee, 2011; Cui et al., 2012; Poloni and Smit, 2012; Go et al., 2013; Zhao et al., 2016). Such CO_2_ adsorption by the tetrazoles can be attributed to hydrogen bonding between CO_2_ molecules and polarizing groups in the tetrazole (Poloni and Smit, 2012; Zhao et al., 2016). We believe that it is this polarizing effect in our (cylopentadienyl)irirdium(III) tetrazole complexes that makes them very good catalysts for CO_2_ hydrogenation catalysts.

Another attribute of the (cylopentadienyl)irirdium(III) tetrazole complexes as CO_2_ hydrogenation catalysts is their chirality at the iridium center. There are prominent examples of optically active half-sandwich piano stool complexes that are chiral-at-metal complexes where the configuration may be stable or labile in solution. Such compounds allow the monitoring of the stereochemical course of substitution reactions as well as being used in organic synthesis in ligand transformation reactions as a result of their chirality (Brunner, 2001). Complexes with chirality at the metal center have been used as effective catalysts in the enantioselective Friedel–Crafts addition of indoles to α,β-unsaturated 2-acyl imidazoles (Huo et al., 2014) and trichloromethylation of 2-acyl imidazoles and 2-acyl pyridines (Huo et al., 2015). In addition, these chiral complexes have been used as hydrogenation catalysts in the hydrogenation of acyclic aromatic N-aryl imines (Imamoto et al., 2006), α-dehydroamino esters, enamides, dimethyl itaconate (Kurihara et al., 2009), and imines (Murata et al., 1999), and in transfer hydrogenation of β,β′-disubstituted nitroalkenes (Chen et al., 2013) and ketones (Tian et al., 2016) with high enantioselectivities and at low catalyst loadings. Despite the impressive array of hydrogenation reactions these complexes are capable of, they have rarely been evaluated as catalysts for CO_2_ hydrogenation. Knowing the role of proton-responsive groups on hydrogenation catalysts and the electron-rich nature along with CO_2_ affinity of tetrazole compounds led to the present study in search of highly active CO_2_ hydrogenation catalysts.

## Experimental

All reactions were carried out in air unless otherwise stated. All solvents used were reagent grade, purchased from Sigma-Aldrich, and dried under nitrogen before use. 1-Phenyl-1*H*-tetrazole-5-thiol, chlorodiphenylphosphine, chlorobis(3,5-dimethylphenyl)phosphine, bis(3,5-di(trifluoromethyl)phenyl)chloro phosphine, and sodium methoxide were purchased from Sigma-Aldrich and used without further purification. Iridium(III) chloride was purchased from Heraeus South Africa and used as received. [Ir(C_5_Me_5_)Cl_2_]_2_ (White et al., 1992) was synthesized according to literature procedures. NMR spectra were recorded on a Bruker 400-MHz NMR spectrometer (^1^H at 400 MHz, ^13^C{^1^H} at 100 MHz, and ^13^P{^1^H} NMR 161.99 MHz). Spectrometer chemical shifts were reported relative to the internal standard tetramethylsilane (δ 0.00 ppm) and referenced to the residual proton and carbon signals at 7.24 and 77.0 ppm, respectively, of CDCl_3_. Infrared spectra were obtained neat using a Perkin Elmer Spectrum BX II fitted with an ATR probe. Melting points were obtained using a Gallenkamp Digital Melting-point Apparatus 5A 6797. Elemental analysis was performed on a Thermos Scientific FLASH 2000 CHNS-O Analyzer. Mass spectrometry was performed using Waters Synapt G2 mass spectrometer with both ESI positive and Cone Voltage 15 V. XRD spectra were obtained from a Bruker APEX-II CCD Diffractometer.

### Synthesis of 5-((bis(3,5-Dimethylphenyl)Phosphino)Thio)-1-Phenyl-1H-Tetrazole (1)

Into a Schlenk tube containing 10-ml THF solution of 50 mg (0.281 mmol) 1-Phenyl-1*H*-tetrazole-5-thiol, 16.6 mg (0.31 mmol) NaOMe was added and the mixture stirred under argon at 0–5°C for 1 h. This was then followed by the dropwise addition of 77.5 mg (0.28 mmol) chlorobis(3,5-dimethylphenyl)phosphine. The reaction mixture was then stirred at room temperature for 16 h. After the reaction time had elapsed, the reaction mixture was filtered via canula and the filtrate dried *in vacuo* to yield the product. Appearance white solid (Yield = 91 mg, 78%). Solubility: Soluble in chloroform, DCM, DMSO; FT-IR (*v*_*max*_*/*cm^−1^): 1,685.85 (C=N). Melting point range: 91–92°C. ^1^H NMR (CDCl_3_, 30°C, δ-ppm): 7.89 (d, ^3^*J* = 12 Hz, 2H, H_arom_), 7.62–7.58 (m, 4H, H_arom_), 7.15 (s, 2H, H_arom_); 2.28 (s, 12H, H_CH3_); ^13^C{^1^H} NMR (CDCl_3_, 30°C, δ-ppm): 155.4, 136.21, 135.29, 134.99, 133.62, 133.44, 133.22, 133.08, 132.84, 132.54 132.07, 131.89, 129.96, 129.74, 129.52, 128.94, 128.77, 128.44, 124.20, 21.70 ^31^P{^1^H} NMR (CDCl_3_, 30°C, δ-ppm): 22.87; HR-ESI-MS [M-Ph]^+^ = 341.0990; Elemental analysis; Anal. calcd. for C_23_H_23_N_4_PS: C, 66.01%; H, 5.54%, N, 13.39% S, 7.66%; Found C, 66.25%; H, 5.18%; N, 13.77%; S, 7.88%.

### Synthesis of 5-((Diphenylphosphino)Thio)-1-Phenyl-1H-Tetrazole (2)

Into a Schlenk tube containing 10-ml THF solution of 500 mg (2.81 mmol) 1-phenyl-1*H*-tetrazole-5-thiol, 166.7 mg (3.086 mmol) NaOMe was added and the mixture stirred under argon at 0–5°C for 1 h. This was then followed by the dropwise addition of 0.50 ml (2.81 mmol) of chlorodiphenylphosphine. The reaction mixture was then stirred at room temperature for 16 h. After the reaction time had elapsed, the reaction mixture was filtered via canula and the filtrate dried *in vacuo* to yield the product. Appearance colorless oil (Yield = 720 mg, 71%). Solubility: Soluble in chloroform, DCM, methanol, DMSO; FT-IR (*v*_*max*_*/*cm^−1^): 1,591.30 (C=N). ^1^H NMR (CDCl_3_, 30°C, δ-ppm): 7.88 (d, ^3^*J* = 7.6 Hz, 4H, H_arom_), 7.73–7.47 (m, 11H, H_arom_); ^13^C{^1^H} NMR (CDCl_3_, 30°C, δ-ppm): 163.67, 133.91, 133.77, 133.59, 132.52, 132.44, 132.32, 132.18, 131.54, 131.34 130.07, 129.96, 129.38, 129.02, 128.80, 128.68, 128.54, 124.19, 124.09 ^31^P{^1^H} NMR (CDCl_3_, 30°C, δ-ppm): 18.54 HR-ESI-MS; [M+O]^−^ = 377.0410; [M-PPh_2_]^−^ = 177.0222; Elemental analysis; Anal. calcd. for C_19_H_15_N_4_PS: C, 62.97%; H, 4.17%; N, 15.46%; S, 8.85%; Found C, 62.46%; H, 4.51%; N, 15.88%; S, 9.01%.

### Synthesis of 5-((bis(3,5-bis(Trifluoromethyl)Phenyl)Phosphino)Thio)-1-Phenyl-1H-Tetrazole (3)

Into a Schlenk tube containing 10-ml THF solution of 50 mg (0.281 mmol) 1-phenyl-1*H*-tetrazole-5-thiol, 16.6 mg (0.31 mmol) NaOMe was added and the mixture stirred under argon at 0–5°C for 1 h. This was then followed by the dropwise addition of 138.21 mg (0.28 mmol) bis(3,5-di(trifluoromethyl)phenyl)chlorophosphine. The reaction mixture was then stirred at room temperature for 16 h. After the reaction time had elapsed, the reaction mixture was filtered via canula and the filtrate dried *in vacuo* to yield the product. Appearance white solid (Yield = 148 mg, 83%). Solubility: Soluble in chloroform, DCM, DMSO; FT-IR (*v*_*max*_*/*cm^−1^): 1,684.85 (C=N). Melting point range: 95–96°C. ^1^H NMR (CDCl_3_, 30°C, δ-ppm): 8.43–8.40 (m, 4H, H_arom_), 8.26 (s, 2H, H_arom_), 7.85 (m, 2H, H_arom_); 7.58 (m, 2H, H_arom_); ^13^C{^1^H} NMR (CDCl_3_, 30°C, δ-ppm): 161.29, 134.21, 134.09, 133.99, 133.52, 133.44, 133.32, 133.08, 132.54, 131.54 131.07, 130.00, 129.96, 129.44, 129.12, 128.90, 128.77, 128.54, 124.19, ^31^P{^1^H} NMR (CDCl_3_, 30°C, δ-ppm): 13.30 HR-ESI-MS; [M]^+^ = 634.5455; Elemental analysis; Anal. calcd. for C_23_H_11_F_12_N_4_PS: C, 43.55%; H, 1.75%, N, 8.83%; S, 5.05%; Found C, 43.46%; H, 1.53%; N, 9.02%; S, 4.99%.

### Synthesis of [IrCp^*^Cl_2_(P(Ph)_2_OH)] (5)

Into a Schlenk tube, 86 mg (0.24 mmol) 5-((diphenylphosphino)thio)-1-phenyl-1H-tetrazole (**2**) was added to 95 mg (0.12 mmol) of [*Ir*(*C*_5_*Me*_5_)*Cl*_2_]_2_ using 5.00 ml of methanol as solvent. The reaction mixture was then stirred for 24 h under argon at room temperature. After the reaction time had elapsed, the precipitate formed was filtered off in air, and the filtrate dried with the crude product obtained being washed with 10 ml 1:1 (v:v diethylether: hexane) and subsequently dried *in vacuo* for 6 h to yield **(5)**. Appearance: Orange solid, (Yield = 94 mg, 52%). Solubility: Soluble in chloroform, DCM, methanol; FT-IR (*v*_*max*_*/*cm^−1^): 3,050.47 (s, P-OH). Melting point range: 167°C (Decomp); ^1^H NMR (CDCl_3_, 30°C, δ-ppm): 7.67–7.62 (m, 9H, H_arom_), 7.44–7.24 (m, 6H, H_arom_), 1.46 (s, 15H, ${\text{H}}_{\text{C}{\text{p}}^{*}}$); ^13^C{^1^H} NMR (CDCl_3_, 30°C, δ-ppm): 132.54; 132.42; 131.41; 128.01; 127.0; 127.84; 93.01; 9.28; ^31^P{^1^H} NMR (CDCl_3_, 30°C, δ-ppm) = 76.92; HR-ESI-MS [M-Cl]^+^ = 565.1045; Elemental analysis; Anal. calcd. for C_22_H_26_Cl_2_IrOP: C, 44.00%; H, 4.36%; Found C, 44.46%; H, 4.51%.

### Synthesis of [IrCp^*^Cl(P(Ph)_2_OH)S-Tz] (8)

The residue obtained from the procedure above in the synthesis of **5** was washed with 10 ml 1:1 (v:v diethylether: hexane) and dried *in vacuo* to yield (**8**). Appearance: Yellow solid, (Yield = 77 mg, 43%). Solubility: Soluble in chloroform, DCM; FT-IR (*v*_*max*_*/*cm^−1^): 2,968.50 (s, P-OH), 1,499.68 (C=N). Melting point range: 218°C (Decomp); ^1^H NMR (CDCl_3_, 30°C, δ-ppm): 8.05 (m, 2H, H_arom_), 7.80 (m, 2H, H_arom_), 7.64 (m, 2H, H_arom_), 7.44 (m, 6H, H_arom_), 7.14 (m, 3H, H_arom_), 1.40 (s, 15H, ${\text{H}}_{\text{C}{\text{p}}^{*}}$); ^13^C{^1^H} NMR (CDCl_3_, 30°C, δ-ppm): 133.64; 133.55; 133.40; 133;20; 132.91; 132.78; 132.68; 132.42; 132.12; 131.31; 129.09; 128.44; 127.84; 96.41; 9.48; ^31^P{^1^H} NMR (CDCl_3_, 30°C, δ-ppm) = 56.83; HR-ESI-MS [M-Cl]^+^ = 707.1599; Elemental analysis; Anal. calcd. for C_29_H_31_ClIrN_4_OPS: C, 46.92%; H, 4.21%, N, 7.55%, S, 4.31%; Found C, 47.11%; H, 4.61%; N, 7.82%; S, 4.12%.

### Synthesis of [IrCp^*^Cl(P(Ph-CF_3_)_2_OH)S-Tz] (9)

Into a Schlenk tube, 70 mg (0.11 mmol) of 5-((bis(3,5-bis(trifluoromethyl)phenyl)phosphino)thio)-1-phenyl-1H-tetrazole **(3)** was added to 44 mg (0.05 mmol) of [Ir(C_5_Me_5_)Cl_2_]_2_ using 5.00 ml of CHCl_3_ as solvent. The reaction mixture was then stirred for 24 h at room temperature. After the reaction time had elapsed, the solvent was dried with the crude product obtained being washed with 10 ml 1:1 (v:v diethylether: hexane) and subsequently dried *in vacuo* for 6 h to yield **(9)**. Appearance: Orange solid (Yield = 37 mg, 67%). Solubility: Soluble in chloroform, DCM, THF; FT-IR (*v*_*max*_*/*cm^−1^): 2,974 (s, P-OH), 1,617.72 (C=N). Melting point range: 192°C (Decomp); ^1^H NMR (CDCl_3_, 30°C, δ-ppm): 8.63–8.40 (m, 4H, H_arom_), 8.31 (s, 2H, H_arom_), 7.85 (m, 2H, H_arom_); 7.63 (m, 2H, H_arom_); ^13^C{^1^H} NMR (CDCl_3_, 30°C, δ-ppm): 160.11, 135.99, 134.69, 133.82, 133.52, 133.44, 133.08, 132.99, 132.42, 131.54, 131.07, 130.00, 129.96, 129.44, 129.36, 129.00, 128.66, 128.00, 123.01, 96.52, 9.43; ^31^P{^1^H} NMR (CDCl_3_, 30°C, δ-ppm) = 55.15; HR-ESI-MS [M-Cl]^+^ = 979.1093; Elemental analysis Anal. calcd. for C_33_H_27_ClF_12_IrN_4_OPS: C, 39.08%; H, 2.68%, N, 5.52%, S, 3.16%; Found C, 40.12%; H, 3.01%; N, 5.61%; S, 3.33%.

### General CO_2_ Hydrogenation Reaction

Into a stainless steel reactor, 1 μmol of an appropriate catalyst was dissolved in 10 ml of appropriate solvent. 1.44 mmol of the appropriate base was added and the resulting solution was pressurized with the required CO_2_:H_2_ ratios. The reaction mixture was then stirred at the appropriate temperature for the required time at 1,500 rpms. After the reaction time had elapsed, the reactor was cooled and the gasses slowly vented. 0.4 ml of the contents of the reactor was sampled and added to 0.2 ml of D_2_O and 5 μl of DMF (as standard). Amounts of product (formate) obtained was calculated by integral relations between singlet formate peak at 8.3 ppm and the singlet DMF formamide peak at 7.9 ppm in the ^1^H NMR spectrum of the sample.

## Synthesis and Characterization of Tetrazole-Based Ligands 1–3

**Scheme 1:**
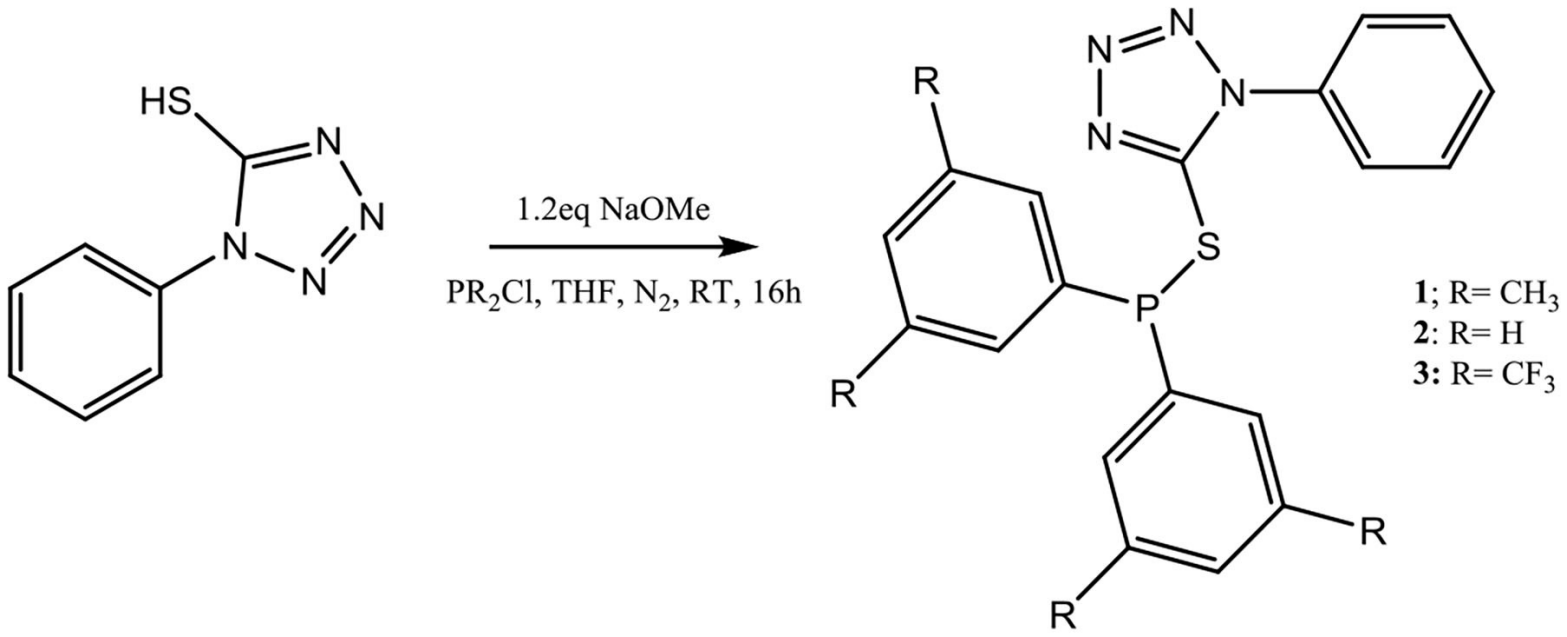
Synthesis of thio-tetrazole ligands **1–3**.

Synthesis of three thio-tetrazole compounds **1**–**3** (Scheme 1) was achieved by first reacting 1-phenyl-1*H*-tetrazole-5-thiol with a slight excess of NaOMe, to deprotonate the hydrogen in the thiol functional group, followed by the addition of the appropriate chlorodialkylphosphine and subsequent separation of NaCl. All three compounds were isolated in excellent yields as colorless oil for **2**, and as whitish solids for **1** and **3** that are air and moisture sensitive. They are soluble in most organic solvents (such as chloroform and dichloromethane) and were characterized by NMR spectroscopy. The easiest way to monitor this reaction was by^31^P{^1^H} NMR spectroscopy. The phosphorus peak for chlorodiphenylphosphine is around 85 ppm, while the peaks for compounds **1**, **2**, and **3** are 22.9 ppm, 18.0 ppm, and 13.3 ppm, respectively. To confirm that compounds **1**–**3** were unoxidized, we prepared the oxidized forms of **1**–**3**, which have phosphorus peaks at 29 ppm, 31.0 ppm, and 25 ppm, respectively. Mass spectra of the ligands also confirmed their formation for which molecular ions or masses of fragmented moieties are observed (Supplementary Figures 15–17).

## Synthesis and Characterization of Tetrazolyl Iridium Complexes

**Scheme 2:**
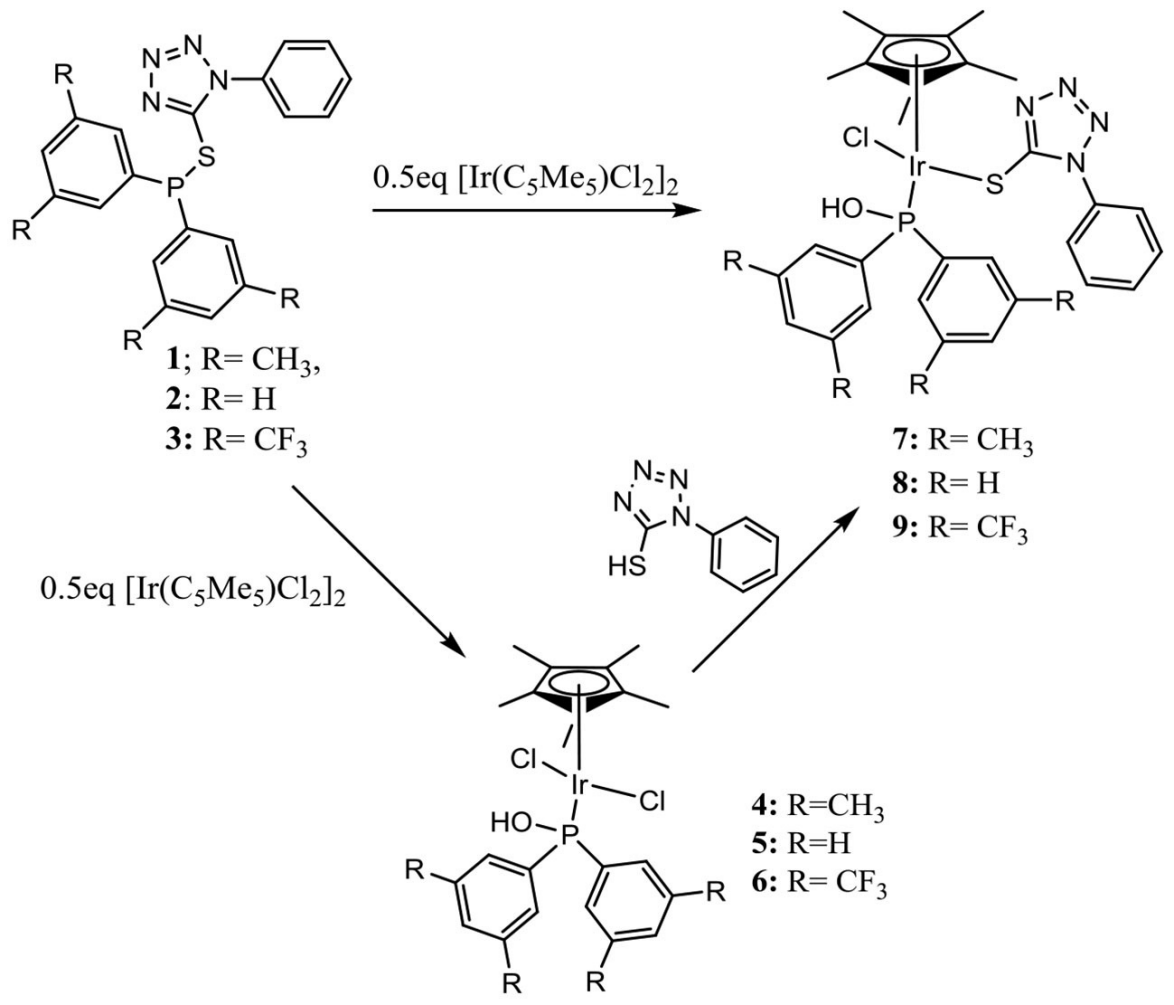
Synthesis of tetrazolyl iridium complexes.

**Figure 1:**
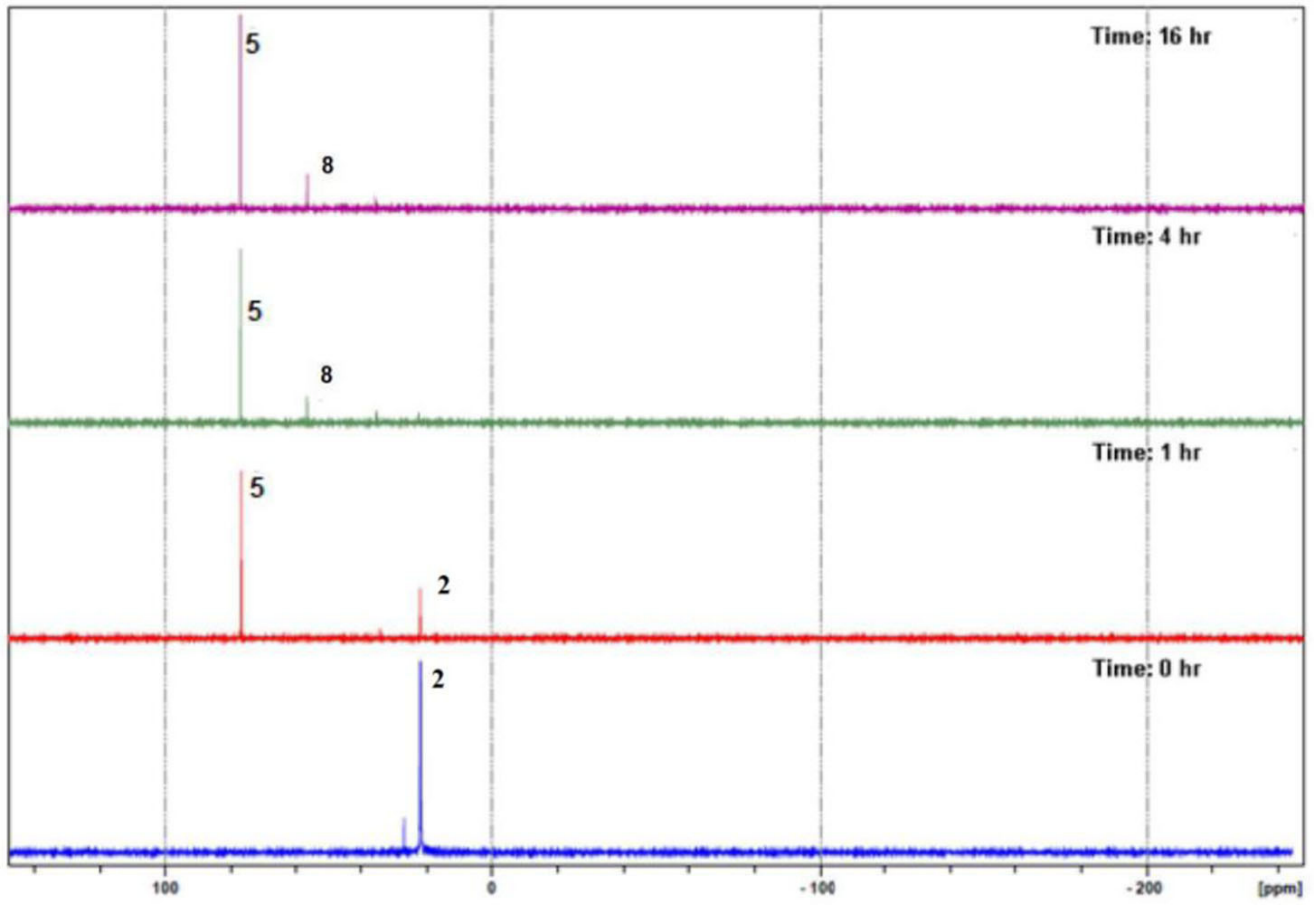
*In situ* ^31^P{^1^H} NMR spectroscopy monitoring of the reaction between **2** and [Ir(C_5_Me_5_)Cl_2_]_2_.

Reacting compounds **1**–**3** with [Ir(C_5_Me_5_)Cl_2_]_2_ in a 2:1 ratio yielded interestingly complexes **4**–**9** (Scheme 2), all of which contain portions of the original compounds used to ligate (C_5_Me_5_)Ir fragment, thus suggesting that the original compounds expected to be the ligand in a product had hydrolyzed. For example, monitoring the reaction between compound **2** and 0.5 eq [Ir(C_5_Me_5_)Cl_2_]_2_ by ^31^P{^1^H} NMR spectroscopy showed that the reaction yielded Iridium **5** within 1 h, and only after 4 h was **8** observed as a second product (Figure 1). It was only after 24 h that the reaction mixture showed complexes **5** and **8** formed in nearly the same amounts, clearly demonstrating that the rate of formation of **8** is much slower than that of **5**. All iridium complexes were obtained as yellow solids in excellent yields and readily soluble dichloromethane and chloroform, but some of these compounds are insoluble in methanol. The structures of these complexes were confirmed by mass spectrometry for which peaks pertaining to [M-Cl]^+^ in addition to the corresponding isotopic peaks are observed. For example, complex **5** is soluble in methanol while complex **8** is insoluble in methanol. Therefore, carrying out the complexation reaction in methanol provides an easy route to separate the different iridium complexes that are formed.

To confirm that the products from the reactions of **1**–**3** with [Ir(C_5_Me_5_)Cl_2_]_2_ are the results of a water-assisted hydrolysis of the P–S bonds present in the ligands, the progress of the reaction between **2** and [Ir(C_5_Me_5_)Cl_2_]_2_ was monitored *in situ* with ^31^P{^1^H} NMR spectroscopy in D_2_O. We observed distinct ^31^P{^1^H} NMR peaks that can be attributed to deuterated and non-deuterated analogs of **5** and **8** (Supplementary Figure 1).

Interestingly, there is very little oxidation of the phosphorus atom in **2** as observed from the *in situ* ^31^P{^1^H} NMR studies in the presence of D_2_O (Supplementary Figure 1). This indicates that the presence of adventitious water in these reactions only hydrolyzes the P–S bond in compound **2** leading to the formation of **5** and subsequently the reaction of **5** with *in situ* generated 1-phenyl-1*H*-tetrazole-5-thiol to form **8**. This reaction route was also confirmed by the reaction of isolated **5** with phenyl-1*H*-tetrazole-5-thiol that forms **8** in <5 min.

Changing the metal precursor from [Ir(C_5_Me_5_)Cl_2_]_2_ to [Ir(C_5_Me_5_)(H_2_O)_3_][SO_4_]^2−^ in the reaction with **2** resulted in the formation of new species where ^13^P{^1^H} NMR peaks at 77 ppm and 57 ppm are observed. Considering **5** and **8** have ^13^P{^1^H} NMR peaks around 77 ppm and 57 ppm, respectively, we propose the formation of aqua/sulfato-**5** at 77 ppm and aqua/sulfato-**8** at 57 ppm where the chloride ligands in **5** and **8** are substituted with aqua or sulfato ligands in each complex. Observing similar P–S heterolysis with different iridium precursors indicated that P–S heterolysis may also be assisted by the presence of the metal. Similar metal-assisted bond hydrolysis has been reported in literature (Ahmad et al., 2019). In order to confirm what effects substituents on the phenyl groups bonded to the phosphorus atom have on coordination mode for the monodentate Iridium(III) complexes, two new ligands with electron-withdrawing (**3**) and electron-donating groups (**1**) on the phenyl groups were reacted with [Ir(C_5_Me_5_)Cl_2_]_2_ (Scheme 2).

**Figure 2:**
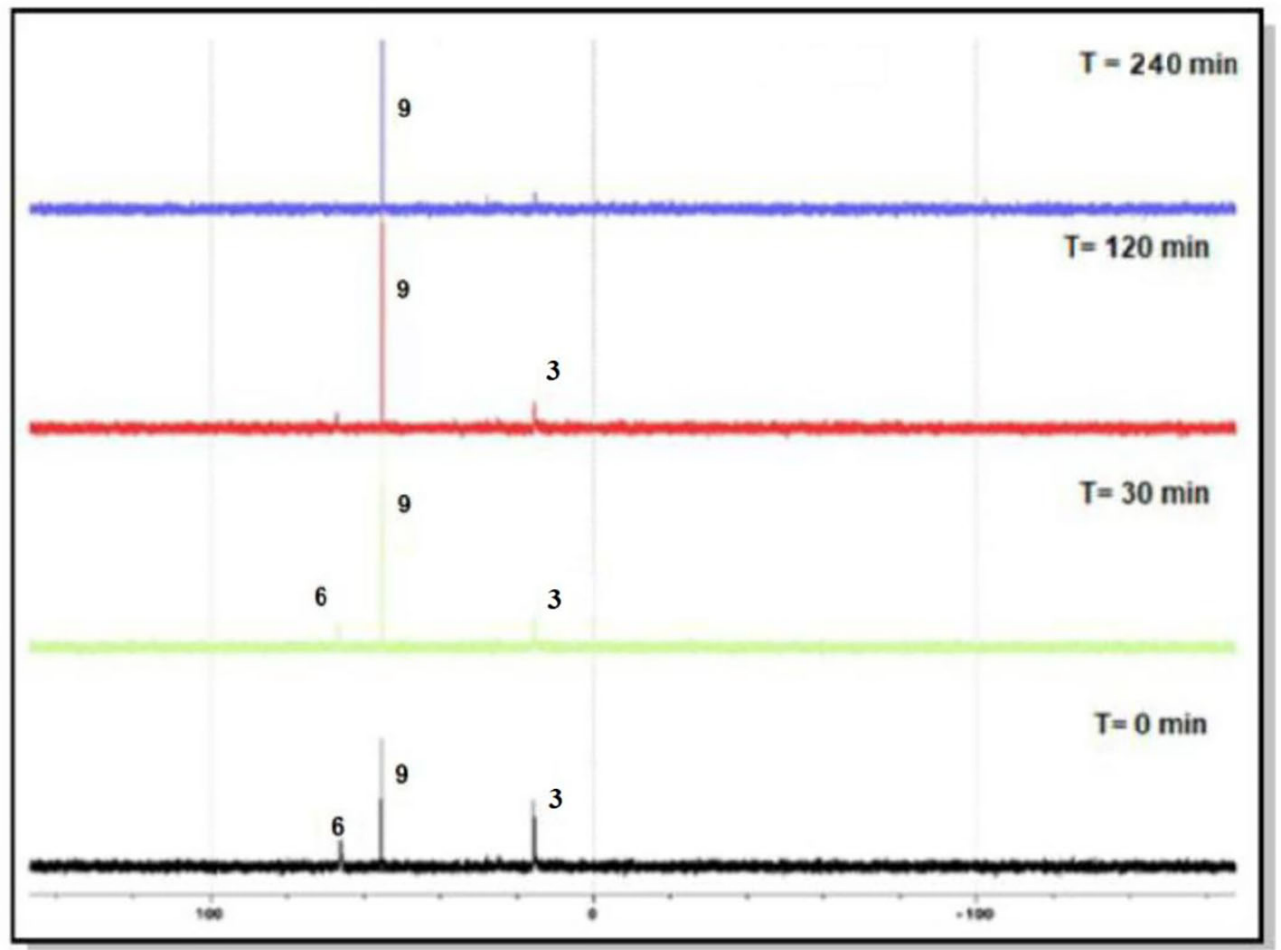
Reaction between **3** and [Ir(C_5_Me_5_)Cl_2_]_2_ monitored by ^31^P{^1^H} NMR spectroscopy.

The progress of each reaction was monitored by ^31^P{^1^H} NMR spectroscopy. Figure 2 shows the changes that occurred in the course of the reaction between **3** and [Ir(C_5_Me_5_)Cl_2_]_2_, where a new Iridium phosphorus intermediate (**6**) with a peak around 65 ppm was converted in less than 2 h to complex **9** with a peak 55 ppm. However, in the case of the reaction of ligand **1** with [Ir(C_5_Me_5_)Cl_2_]_2_, even after heating the reaction mixture at 50°C for 24 h, we observed various phosphine species (Supplementary Figure 2). The phosphorus peak at 77 ppm was assigned to **4**, the peak at 58 ppm was assigned to **7**, but the peak at 33.8 ppm remains unidentified. We were, however, unable to separate the complexes in the reaction mixture as they are all soluble in similar solvents. It is, nevertheless, evident that ligands **1**, **2**, and **3** react with [Ir(C_5_Me_5_)Cl_2_]_2_ in the following order: ligand with electron-withdrawing substituent **>** ligand with no substituent > ligand with electron-donating substituent. This indicates that the electron-withdrawing ability of substituents attached to the phosphorus atom plays an important role in the heterolysis and in subsequent stabilization of iridium-phosphorus in bonds **7**, **8**, and **9**. The latter can be explained by the fact that the π-acid strength of the phosphorus atom follows the order 3,5-(CH_3_)_2_-C_6_H_3_)P(OH) < (C_6_H_5_)P(OH) <3,5-(CF_3_)_2_-C_6_H_3_)P(OH). The electron-withdrawing (CF_3_) group concentrates electron density away from the phosphorus atom, which favors increased π back-donation to the phosphorus, thereby reinforcing the Ir–P bond more in **9** than in **8** and **7**.

## Single Crystal X-ray Structures of Complex 8

**Figure 3:**
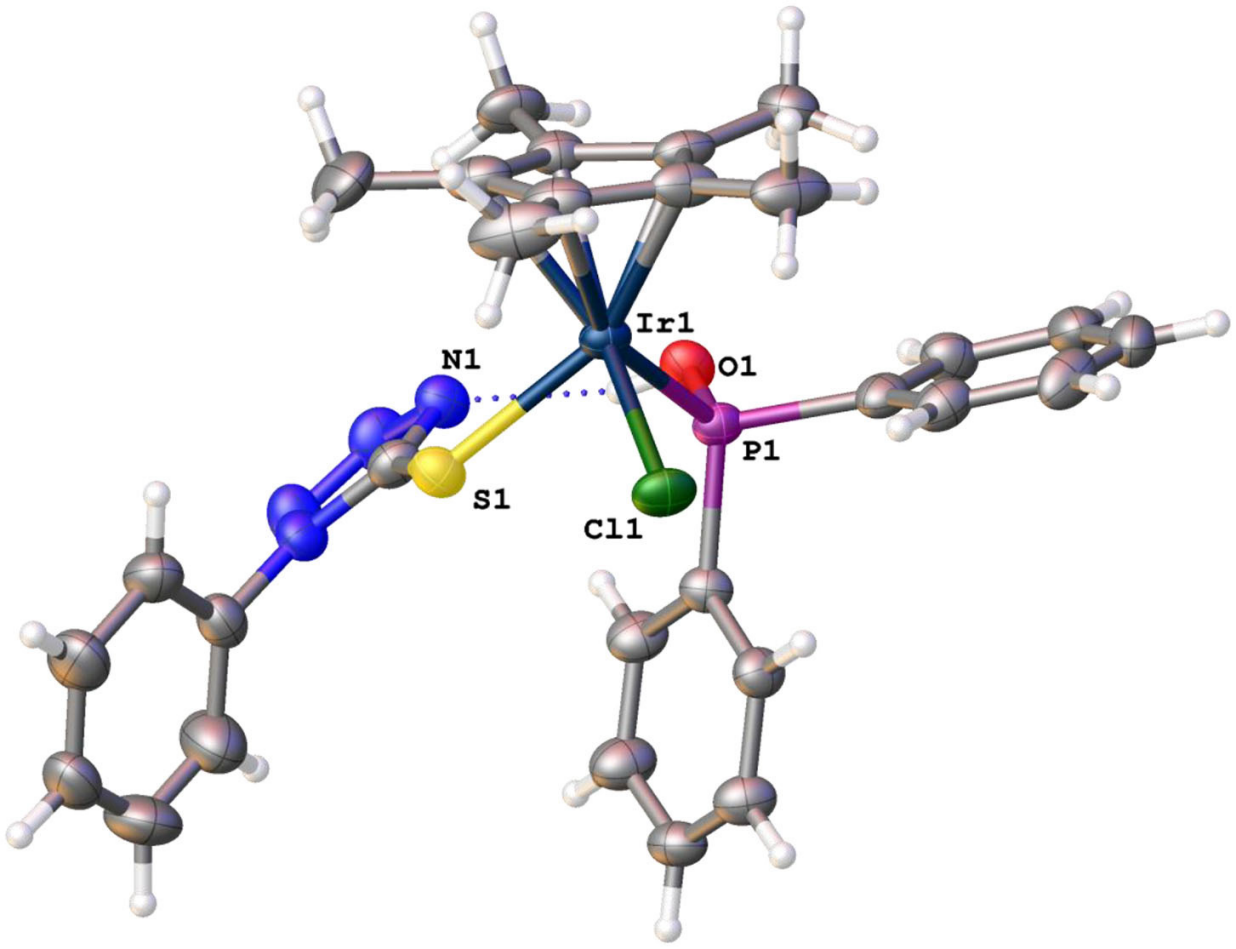
Molecular structures of enantiopure complex **8**.

Enantiopure crystals for **8** were grown by slow evaporation from a solution of **8** in CHCl_3_, and the crystallographic data of this complex are shown in Supplementary Table 2. Complex **8** crystallizes in the monoclinic crystal system with space group P2_1_/n with piano-stool geometry around the iridium center. The iridium center is an iridium(III) center with a negatively charged sulfur moiety as ligand. Interestingly, hydrogen bonding is observed between the hydroxyl protons bonded to the phosphorus atom in complex **8** and one of the nitrogen atoms in the tetrazole ring (Figure 3).

**Figure 4:**
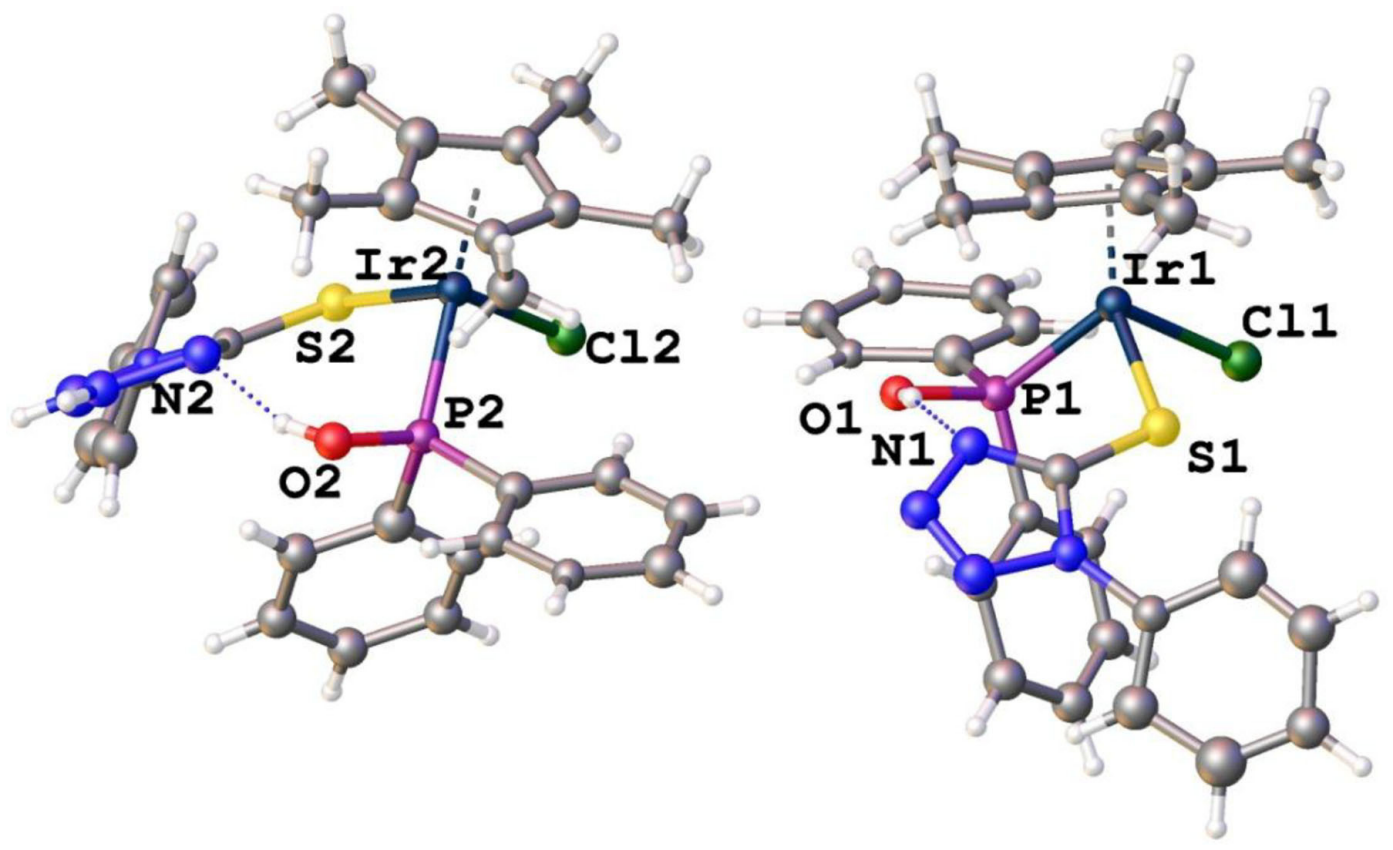
Molecular structures of racemic complex **8** (solvent molecules removed for clarity).

It is interesting to note that re-growing crystals of **8** results in racemic co-crystallization of enantiomers of **8** that have chirality at the metal center in the triclinic space group *P-1*. As expected, the complexes have piano-stool geometry around both iridium centers with a negatively charged sulfur containing moiety as ligand (Figure 4). Similarly, hydrogen bonding is observed between the hydroxyl protons and a nitrogen atom in the tetrazole ring. Supplementary Tables 1, 2 show selected bond lengths and angles and crystallographic data, respectively, for enantiopure complex **8** and racemic complex **8**. The bond angles and distances obtained for racemic and enantiomer **8** are in similar ranges for reported Ir–S or Ir–P complexes (Hughes et al., 2001; Ludwig et al., 2012). The high *R*_int_ = 0.1542 for racemic complex **8** is possibly the result of twinning.

## Evaluation of Chiral-at-Metal Iridium Complexes in CO_2_ Hydrogenation

Using either ethanol or DMSO as solvents for CO_2_ hydrogenation, there was only trace amounts of formate detected in the ^1^H NMR spectrum of the reaction mixture using **5**, **8**, or **9** (the product obtained is a DBU salt of formic acid and was not separated). However, changing the solvent to water resulted in the formation of a modest amount of formate (0.027 mmol) using **8**. This increase in TONs is probably because water has been shown to enhance the rate of CO_2_ hydrogenation possibly due to hydrogen-bonding interaction between water and an oxygen atom in CO_2_ during the insertion of CO_2_ in the catalytically active metal hydride species (Suna et al., 2017). However, adding THF resulted in an eight-fold increase in TON that indicates the importance of THF as solvent for the reaction. Increasing the temperature of the reaction also resulted in an increase in the amount of formate formed. At 160°C, 0.656 mmol of formate was produced compared to 0.197 at 90°C (Table 1). Varying the ratios of both CO_2_ and H_2_ gave the ideal ratio of CO_2_:H_2_ at 40 bar. Changing the ratio of H_2_:CO_2_ from 3:1 to 1:1 generally resulted in an improvement in TONs. This suggests that the rates of formation of the catalytically active iridium hydride species and the insertion of CO_2_ into the iridium–hydride bond proceed at similar rates. Furthermore, addition of different bases affect the TONs of the reaction (Table 1). The best base for the reaction is DBU (Table 1, entries 11 and 12). We also observed that the amount of product formed is higher than the amount of base used. This is possible because 1 eq of DBU is known to stabilize multiple equivalences of formate via homoconjugation during the CO_2_ hydrogenation reaction (Munshi et al., 2002; Getty et al., 2009; Jeletic et al., 2014; Zhang et al., 2015).

**Table 1:** Optimization of reaction conditions for the CO_2_ hydrogenation reaction using **8**.

**Entry**	**Solvent (10 ml)**	**Temperature (^**°**^C)**	**Pressure (H_**2**_: CO_**2**_) (bar)**	**Base (1.44 mmol)**	**mmol Product**	**TON**
1	H_2_O	90	10:30	K_2_CO_3_	0.027	5
2	H_2_O:THF(4:1)	90	10:30	K_2_CO_3_	0.197	39
3	DMSO	90	10:30	K_2_CO_3_	Trace	–
4	Ethanol	90	10:30	K_2_CO_3_	Trace	–
5	H_2_O:THF(4:1)	140	10:30	K_2_CO_3_	0.329	66
6	H_2_O:THF(4:1)	160	10:30	K_2_CO_3_	0.656	131
7	H_2_O:THF(4:1)	160	13.3:26.6	K_2_CO_3_	0.652	130
8	H_2_O:THF(4:1)	160	20:20	K_2_CO_3_	0.744	149
9	H_2_O:THF(4:1)	160	26.6:13.3	K_2_CO_3_	0.576	115
10	H_2_O:THF(4:1)	160	20:20	Et_3_N	0.451	90
11	H_2_O:THF(4:1)	160	20:20	DBU	2.350	470
12	H_2_O:THF(4:1)	160	20:20	DBU	1.561	1,561
13^a^	H_2_O:THF(4:1)	160	20:20	DBU	0.057	281
14^b^	H_2_O:THF(4:1)	160	20:20	DBU	0.704	704

*Reactions were performed in 10 ml of appropriate neat solvent or mixture, 1.44 mmol of base at appropriate temperature and pressure with stirring (1,500 rpm), and complex **8** (5 μmol) for 24 h using DMF as standard. TON = calculated mmol of [HCO${}_{2}^{-}$]/mmol of catalyst. (a) 1 μmol of catalyst used for 24 h; (b) 0.2 μmol of catalyst used in 24-h reaction time; (c) 1 μmol of catalyst used for 16 h*.

Reducing the amount of catalyst used and reaction time resulted in lower amounts of formate produced. As such, the optimum reaction conditions for catalyst **8** in CO_2_ hydrogenation were as follows: 10 ml of a 4:1 mixture of H_2_O:THF, temperature of 160°C, 1:1 mixture of CO_2_:H_2_ at 40 bar, DBU as base, 1 μmol catalyst loading, a 24-h reaction time, and stirring rate of 1,500 rpm. The progress of the reaction could be followed by sampling the reaction mixture after 24 h and running the ^1^H NMR (Supplementary Figure 3) and the ^13^C{^1^H} NMR (Supplementary Figure 4) spectra. The NMR spectra indicated the formation of bicarbonate ion during the course of the CO_2_ hydrogenation, an observation that has also been reported in the literature as a product from CO_2_ and wet DBU (Heldebrant et al., 2005). Hydrogenation of NaHCO_3_ using **8** also resulted in the production of formate (Table 2). This indicates that the hydrogenation of bicarbonate, in addition to CO_2_, is an additional viable route to the production of formate in CO_2_ hydrogenation under the conditions used in our study. Comparing other iridium half-sandwich catalysts with P-OH groups to catalyst **8** (TON 1 561) for CO_2_ hydrogenation, it is evident that catalyst **5** (TON 1 878) performs better than catalyst **8** (Table 2). The performance of these two catalysts indicates that electron-donating substituents on the tetrazolyl results in lower catalytic activity. Nevertheless, it is also possible that increased catalytic activity could be the result of the ease with which chlorides dissociate from the iridium center and the subsequent iridium dihydride species formation. Such secondary hydrido species formation for **8** would be more unfavorable for the following reasons. Since **8** exists as an enantiomer, the P-OH group could play the role of a proton-responsive group, where the enantiomer of **8** with P-OH and chloride ligands oriented away from each other would require a significant amounts of energy in forming the iridium hydride species, but the other enantiomer with both P-OH and Cl groups oriented toward each other would require considerably less energy. Interestingly, catalyst **9**, which has a more electron-deficient iridium center, because of the electron-withdrawing nature of the CF_3_ substituents, also has a similar TON (1,391) to that of **8**. This suggests that the CF_3_ groups are too far from the metal center to significantly influence catalytic activity. This assertion is further supported by ^31^P{^1^H} NMR peaks for both **8** and **9** around 55 ppm, in contrast to more electron-deficient phosphine metal complex peaks that are expected more downfield.

**Table 2:** Chiral-at-metal complexes for CO_2_ hydrogenation.

**Entry**	**Catalyst**	**mmol Product (mmol)^**a**^**	**[${\text{HCO}}_{2}^{-}$] (mM)**	**TON (TON)^**b**^**
1	5	1.878 (1.305)	188	1,878 (1,305)
2	8	1.561 (0.752)	156	1,561 (752)
3	8^c^	0.601	60	601
4	9	1.391 (1.172)	139	1,391 (1,172)
5	[Cp*Ir(L1)(Cl)]Cl^d^	0.26	26	1,300
6	[Cp*Ir(L2)(Cl)]Cl^d^	0.91	91	4,600
7	[Cp*Ir(BiBzImH_2_)Cl]Cl^e^	17.5	350	1,750
8	[Cp*IrCl(NHC-py^OtBU^)]OTf^f^	5.55	222	740
9	[Cp*IrCl(NHC-py^OH^)]OTf^f^	6.45	258	860

*Reactions performed in 10 ml of a 4:1 H_2_O:THF mixture, 0.216 ml DBU at 160°C under 40 bar CO_2_:H_2_ (1:1) with stirring (1,500 rpm) in the presence of a complex (1 μmol) for 24 h. (a) Calculated mmol of [HCO${}_{2}^{-}$] in the presence of excess mercury (500 Hg:1 cat); (b) calculated TON of [HCO${}_{2}^{-}$] when reaction carried out in the presence of excess mercury (500 Hg:1 cat); (c) performed in 10 ml of a 4:1 H_2_O:THF mixture, 1.44 mmol of NaHCO_3_ at 160°C under 50 bar H_2_ with stirring (1,500 rpm) in the presence of a complex (1 μmol) for 24 h. (d) Reference (Suna et al., 2017). **L1** = 2-(1H-imidazol-2-yl)pyridine, **L2** =2-(1H-pyrazol-3-yl)pyridine performed in 10 ml of a 2.0 M KHCO_3_ aqueous solution (pH 8.5) at 50°C under 1.0 MPa H_2_/CO_2_ (1:1) with stirring (1,500 rpm) in the presence of a complex (20 μm) for 24 h. (e) Reference (Gunasekar et al., 2018). The reaction was carried out in a 50-ml 1 M KOH solution of MeOH/H_2_O (1:1) mixture under 4.0 MPa total pressure (CO_2_/H_2_ = 1) using 0.2 mM catalyst at 80°C for 38 h. (f) Reference (Siek et al., 2017). Reactions were performed in 25 ml of an aqueous solution of 0.3 mM catalyst and 1 M NaHCO_3_ at 115°C and 300 psig of H_2_/CO_2_ (1:1) for 18 h*.

When the catalytic activity of **5–9** are compared to other highly active proton-responsive iridium(III) half-sandwich precatalysts for CO_2_ hydrogenation (Siek et al., 2017; Suna et al., 2017; Gunasekar et al., 2018), it is evident that these chiral-at-metal catalysts with proton-responsive P-OH groups are equally as active for CO_2_ hydrogenation as shown by their high TONs. For example, more concentrated formate solutions (around 180 mM) are produced from **5**, **8**, and **9** catalyzed CO_2_ hydrogenation in our study compared to 26–91 mM for N^∧^N bidentate iridium half-sandwich catalysts reported in literature. These concentrated formate solutions are obtained due to higher temperatures and pressures used. However, these catalysts do not require highly basic solutions and/or significantly extended reaction times in order to obtain similar formate solutions.

## Mechanism of Action of Chiral-at-Metal Complexes for CO_2_ Hydrogenation

**Scheme 3:**
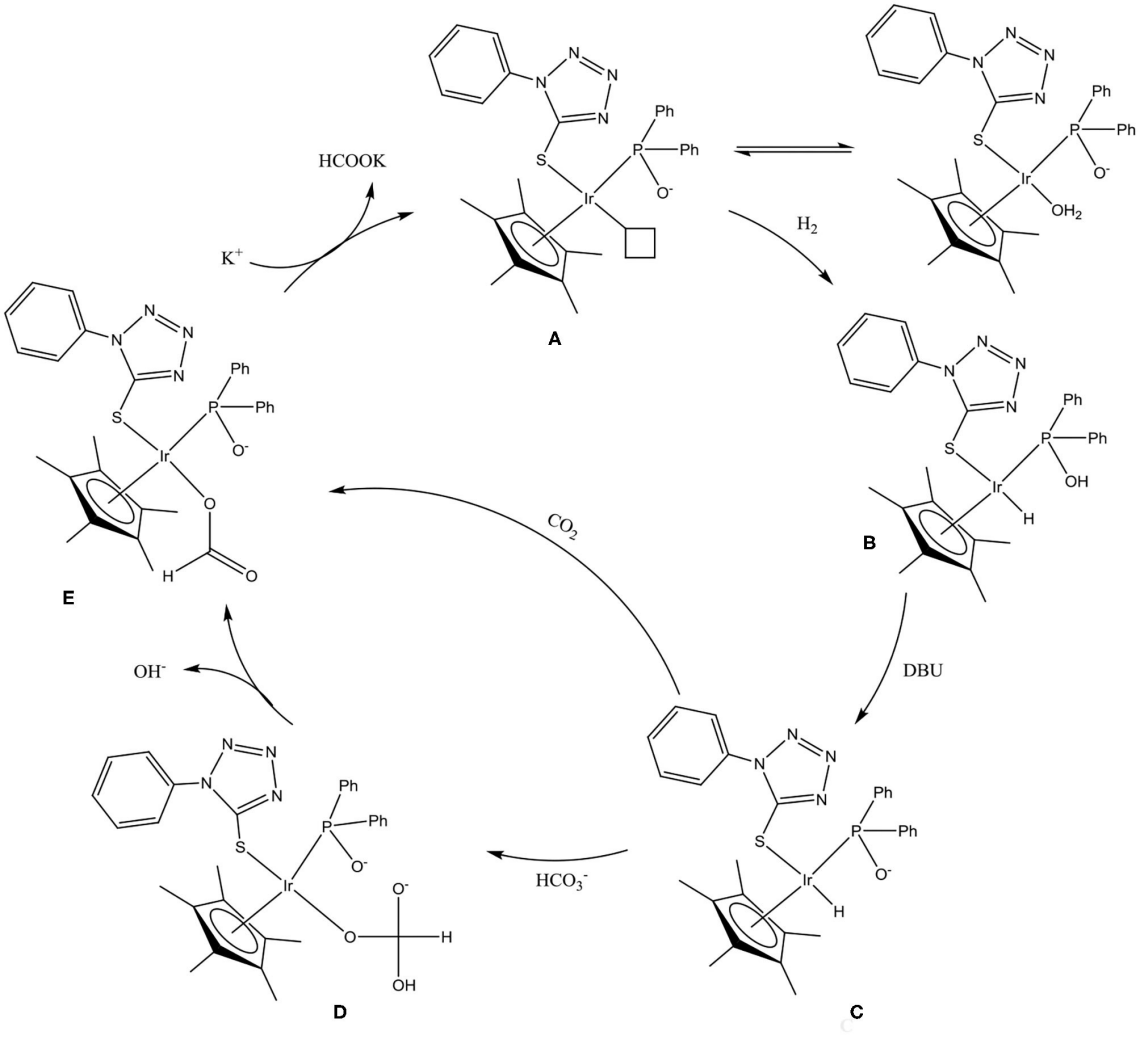
Plausible reaction mechanism for chiral-at-metal Iridium complexes in CO_2_ hydrogenation. **(A)** Deprotonated iridium-aqua complex, **(B)** Iridium-hydride complex, **(C)** Deprotonated iridium-hydride species, **(D)** Iridium-carbonato species, and **(E)** Iridium-formato species.

A mercury drop test indicated that CO_2_ was hydrogenated via a homogeneous/cocktail of catalysts approach (Table 2). As such, plausible reaction mechanisms for the CO_2_ hydrogenation reaction involving the chiral-at-metal Iridium complexes were investigated. In the presence of excess base and water, complex **8** would be expected to undergo an aquation reaction, with the chloro ligand being replaced with a water ligand while deprotonating the OH group on the phosphorus donor occurs to yield **A** (Scheme 3). *In situ* ^1^H NMR experiments involving the reaction of complex **8** with two equivalence of DBU demonstrated the disappearance of the hydroxyl protons at 11.6 ppm indicating deprotonation (Supplementary Figure 5). This indicated that during the hydrogenation of CO_2_, the P-OH group could possibly function as a proton-responsive group to assist in the heterolytic cleavage of H_2_.

**Figure 5:**
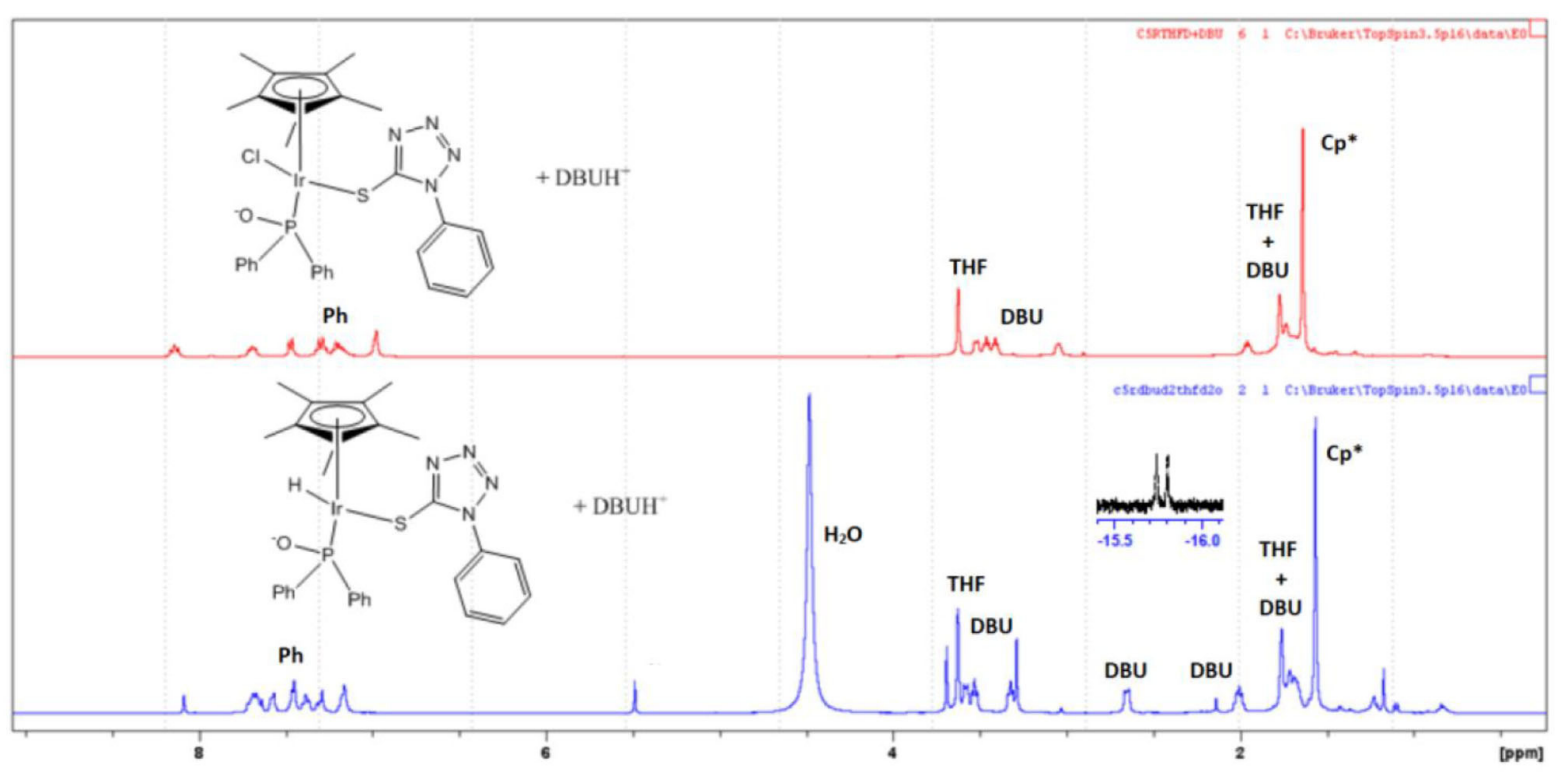
Formation of chiral-at-metal iridium hydride species using **8**.

Pressurizing **8** in a THF/water mixture with two equivalence of DBU and 5 bar of D_2_ at 100°C for 1 h resulted in the appearance of doublet hydride peaks around −15.5 ppm as well as corresponding shifts in peaks in the aromatic region (Figure 5). This was due to the exchange of hydrogen and deuterium present in solution yielding proton signals in the ^1^H NMR spectrum. The observed splitting of hydride signal was as a result of the proximity of the NMR active phosphorus atom. Similar splitting for such hydride protons have also been observed in literature (Feller et al., 2016). In addition, there was a corresponding shift in the ^31^P{^1^H} NMR from around 57 ppm to 72 ppm upon formation of the iridium hydride species (Supplementary Figure 6).

Due to the presence of excess base in solution, the iridium hydride species could be expected to exist as **C** (Scheme 3) since the proton on the P-OH group is deprotonated. Subsequently, insertion of *in situ* generated bicarbonate ions into the iridium–hydride bond yields intermediate **D**, which, upon dissociation of a hydroxyl group, yields the iridium–formato species **E**. However, direct insertion of CO_2_ into the metal–hydride bond is also possible during the formation of the iridium–formato species as a result of the observation of formate when other non-carbonate bases are used for the reaction. Introducing CO_2_ to a solution of **C** results in the disappearance of the hydride signal. Such direct CO_2_ insertion in metal–hydride bonds have been reported in mechanisms involving interactions between CO_2_ and the metal–hydride bonds prior to the formation of the metal–formato species (Wang et al., 2012; Suna et al., 2017). The final step of the reaction is then reductive elimination of formate using the base in order to regenerate species **A** (Scheme 3).

## Conclusion

Unintended iridium complexes (**4**–**9**) were obtained as a result of the heterolysis of P–S tetrazolyl ligands (**1**–**3**) during complexation with [Ir(C_5_Me_5_)Cl_2_]_2_. The heterolysis is ascribed to the presence of water and electron-withdrawing groups on the phosphorus moiety, which were found to facilitate the heterolysis of the P–S bond in the ligand during complexation with [Ir(C_5_Me_5_)Cl_2_]_2_. The iridium complexes hydrogenate CO_2_ as well as NaHCO_3_ in a 4:1 water:THF solvent mixture, and DBU as abase. A key factor in the activity of these iridium complexes as excellent CO_2_ hydrogenation catalysts is the ability to be proton responsive. We observed such proton-responsive enhanced catalytic ability from chiral-at-the-metal complexes that have the P-OH proton-responsive functional groups. Complex **8** was used to establish that the mechanism of the CO_2_ hydrogenation reaction for the iridium complexes proceeded with the iridium precatalysts first undergoing aquation, followed by H_2_ heterolysis that is driven by proton-responsive P-OH groups to form an iridium hydride intermediate. This iridium hydride species is the catalytically active species that hydrogenates either bicarbonate species or CO_2_ to yield the formate product.

## Data Availability Statement

The raw data supporting the conclusions of this article will be made available by the authors, without undue reservation. The crystallographic datasets presented in this study can be found in online repositories. The names of the repository/repositories and accession number(s) can be found below: Cambridge Crystallographic Data Center, CCDC numbers 1840199 and 1993265.

## Author Contributions

JD and BM conceptualized the research. EO designed and carried out the experiments with guidance from BM and JD. EO, JD, and BM discussed and analyzed the experimental data and finalized the manuscript. All authors contributed to the article and approved the submitted version.

## Conflict of Interest

The authors declare that the research was conducted in the absence of any commercial or financial relationships that could be construed as a potential conflict of interest.

## Supplementary Material

Electronic supporting information (ESI) is available free of charge. CCDC number **1840199** contains the supplementary crystallographic data for racemic complex **8**. CCDC number 1993265 contains the supplementary crystallographic data for enantiopure complex **8**. These data can be obtained free of charge from the Cambridge Crystallographic Data Center from the Director, CCDC, 12 Union Road, Cambridge, CB2 1EZ, UK (fax: +44 1223 336063; deposit@ccdc.cam.ac.uk or https://www.ccdc.cam.ac.uk/structures/).
