# Supplementary material for: Chiral-at-Metal: Iridium(III) Tetrazole Complexes With Proton-Responsive P-OH Groups for CO2 Hydrogenation
Source: Front Chem. 2020 Nov 13;8:591353. doi: 10.3389/fchem.2020.591353 (PMC7692406; doi:10.3389/fchem.2020.591353)
Supplement: Supplementary file 1 [file Data_Sheet_5.PDF]

Chiral-at-metal: Iridium(III) tetrazole complexes with proton responsive P-OH groups for CO_2 hydrogenation

Edward Ocansey[1], James Darkwa[1]* and Banothile C.E. Makhubela[1]*

[1]Research Centre for Synthesis and Catalysis, Department of Chemical Science, University of Johannesburg, Auckland Park, 2006, South Africa.

Supplementary Material

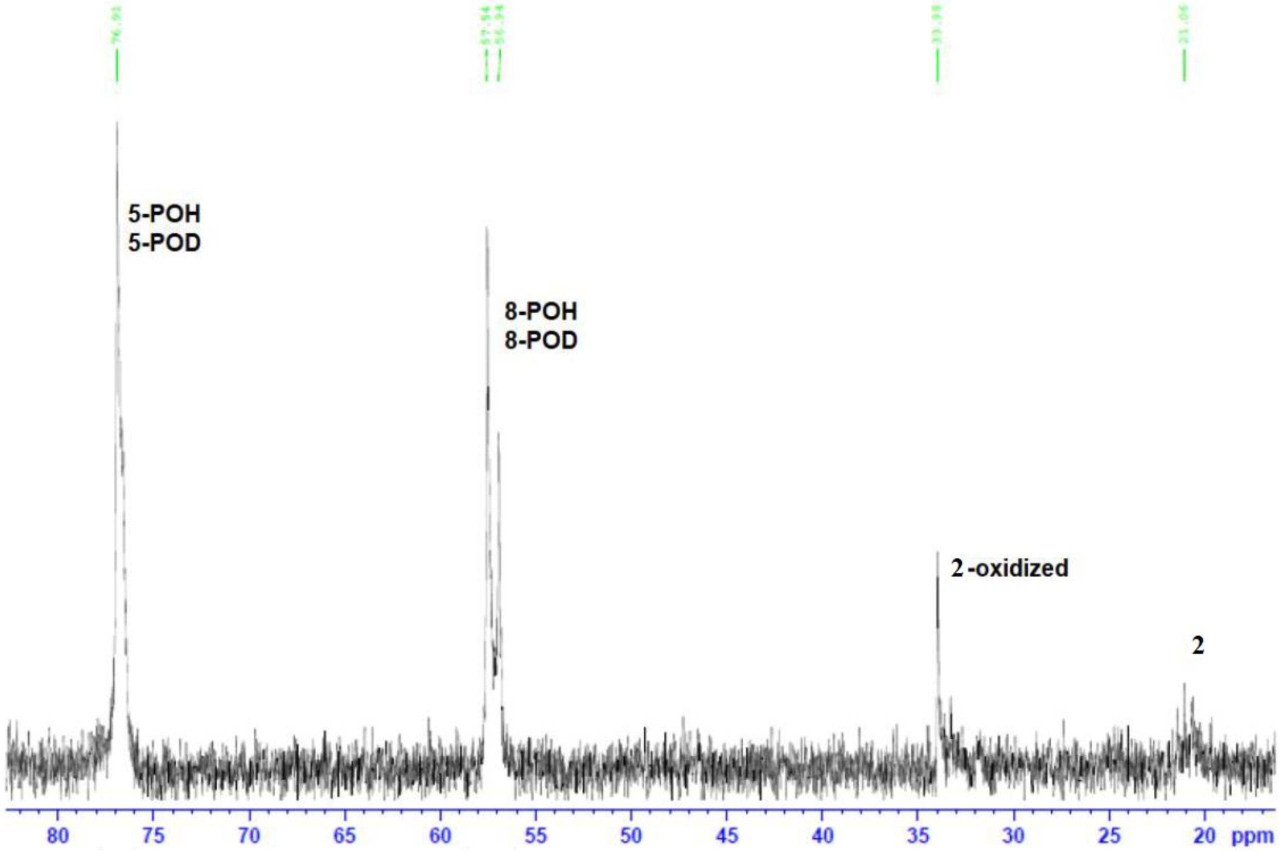

Figure SI-1: $^{31}P\{^1H\}$ NMR spectrum monitoring the reaction between **2** and $[Ir(C_5Me_5)Cl_2]_2$ in the presence of D_2O..

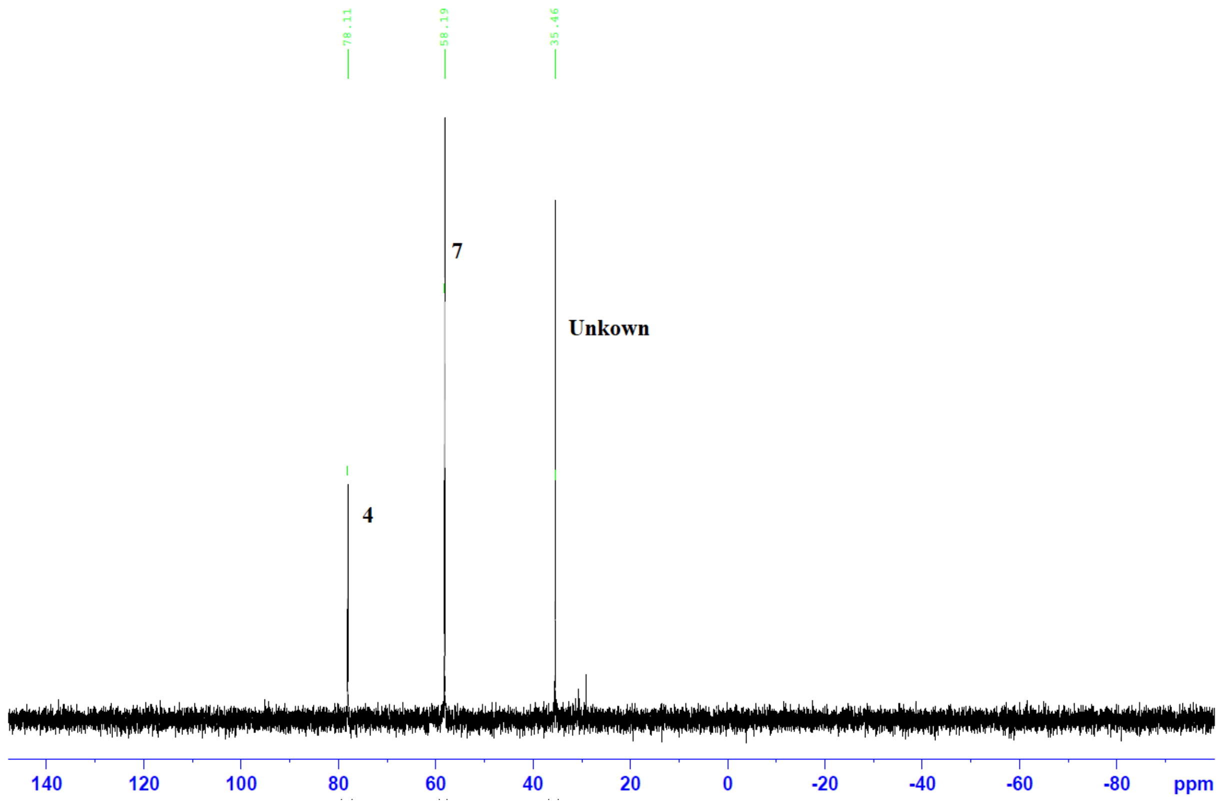

Figure SI-2: $^{31}P\{^{1}H\}$ NMR spectrum monitoring the reaction between **1** and $[Ir(C_5Me_5)Cl_2]_2$

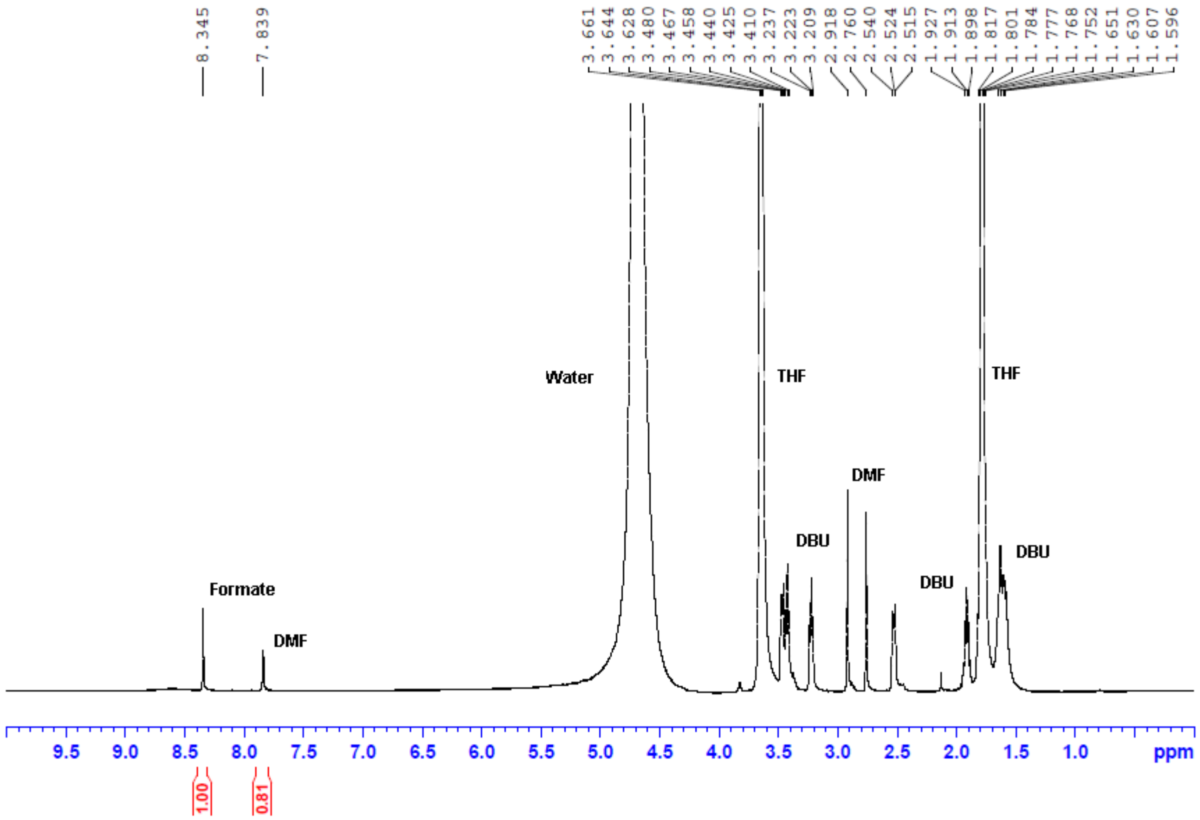

Figure SI-3: The ¹H NMR spectrum the CO_2 hydrogenation reaction using **8** as catalyst, sampled after 24 h with DMF as internal standard.

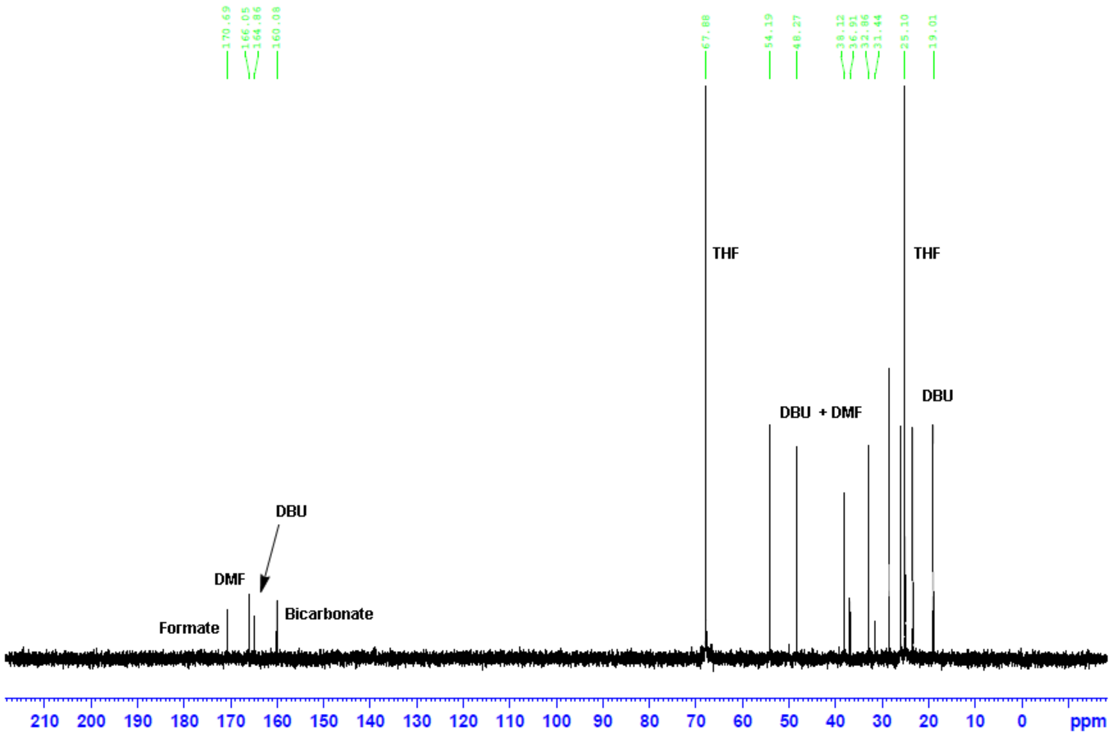

Figure SI-4: The $^{13}C\{^{1}H\}$ NMR spectrum of CO_2 hydrogenation reaction using **8** as catalyst, sampled after 24 h with DMF as internal standard.

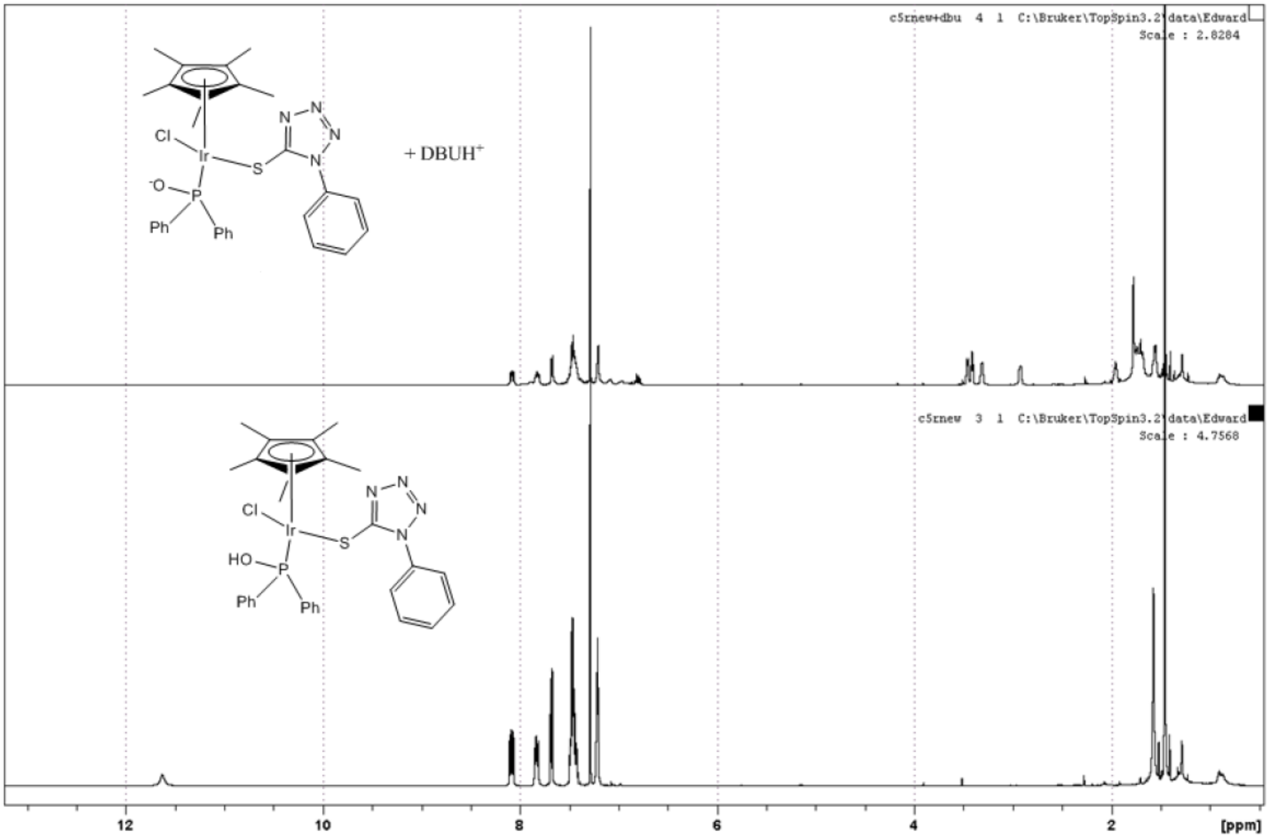

Figure SI-5: Deprotonation of **8** using DBU

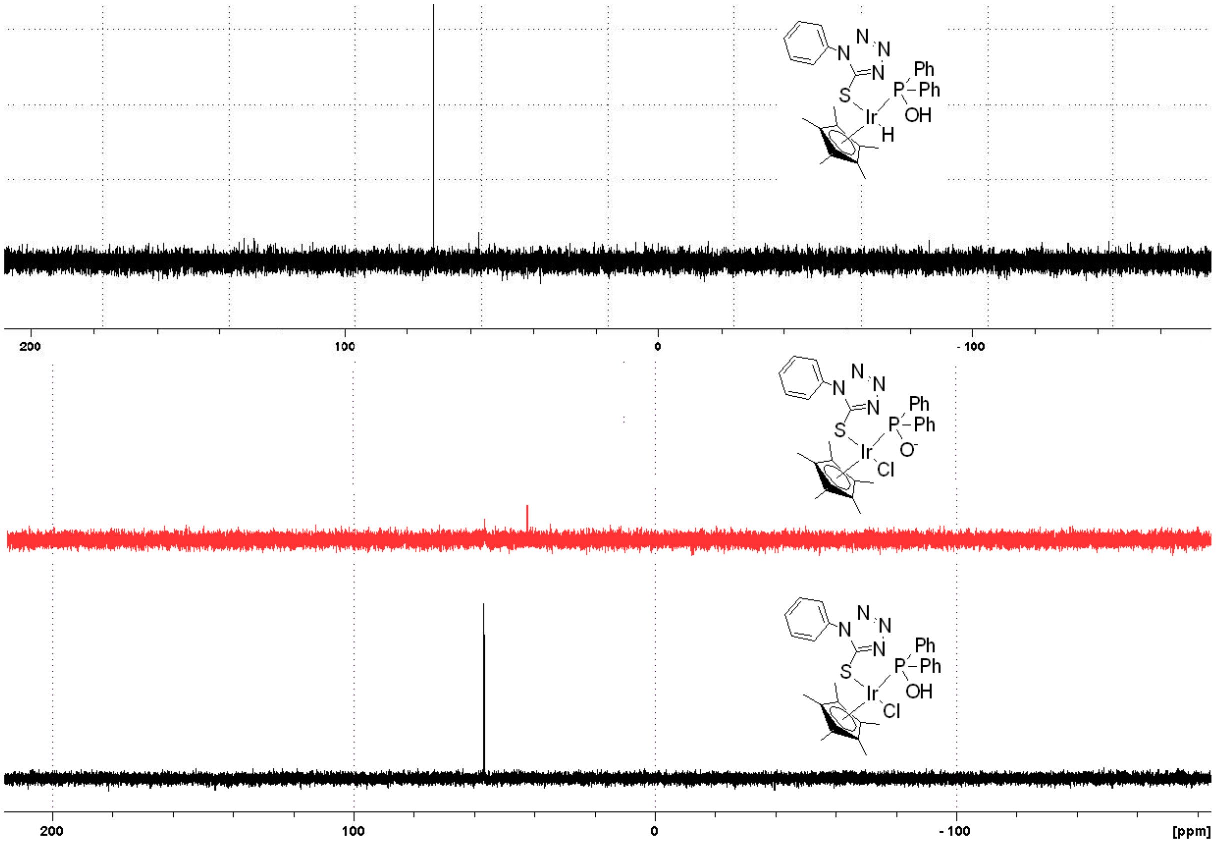

Figure SI-6: *In situ* $^{31}P\{^{1}H\}$ NMR monitoring for the formation of Iridium hydride

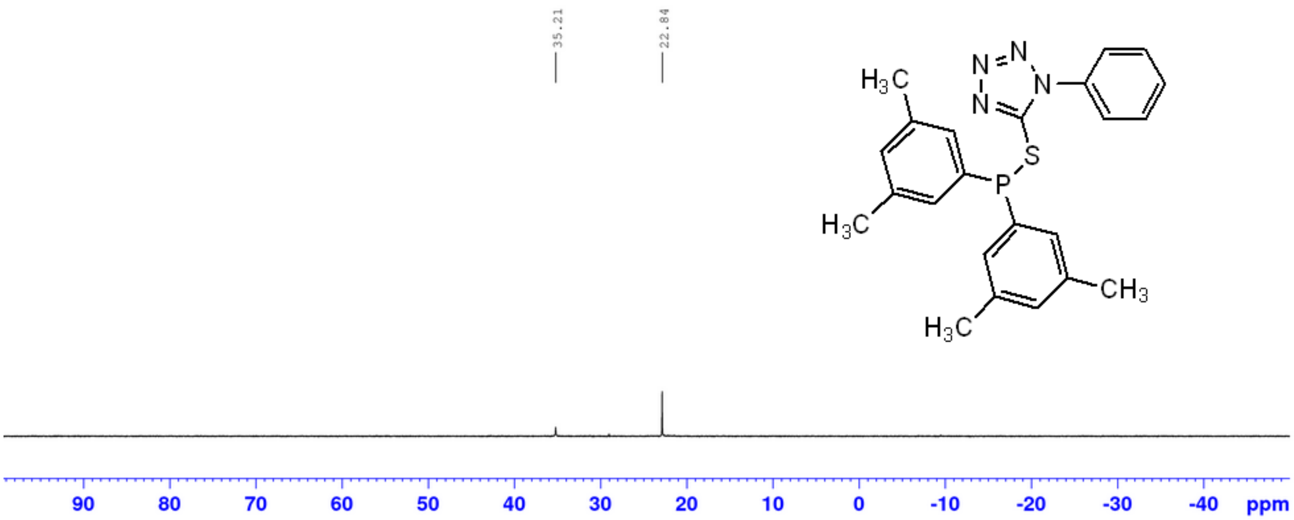

Figure SI-7: $^{31}P\{^{1}H\}$ NMR spectrum of **1**

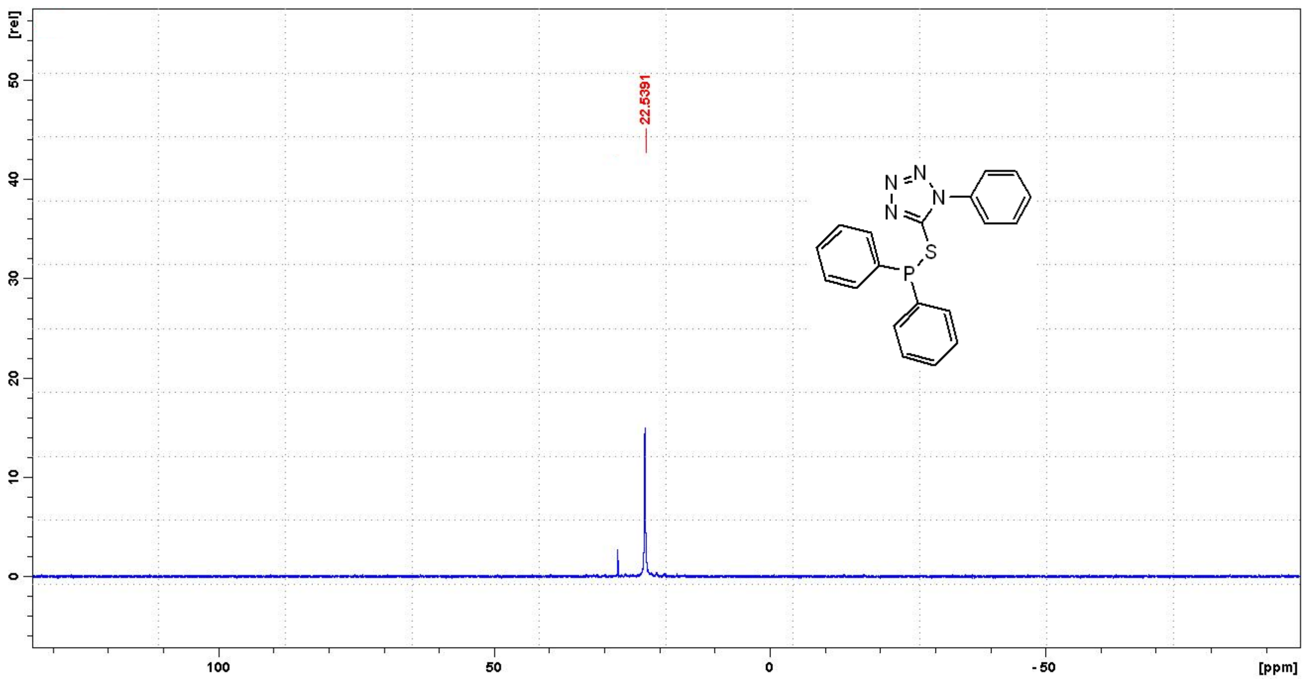

Figure SI-8:$^{31}P\{^1H\}$ NMR spectrum of **2**

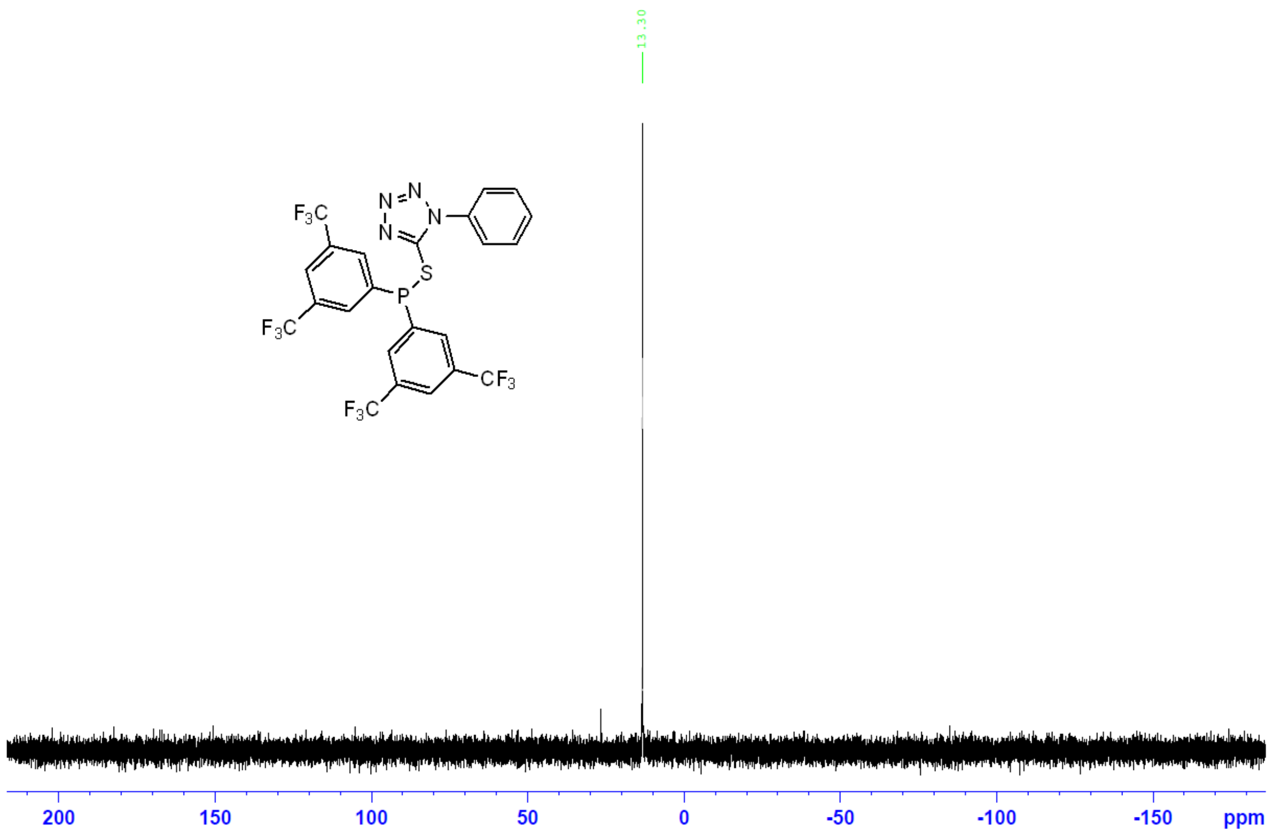

Figure SI-9:$^{31}P\{^1H\}$ NMR spectrum of **3**

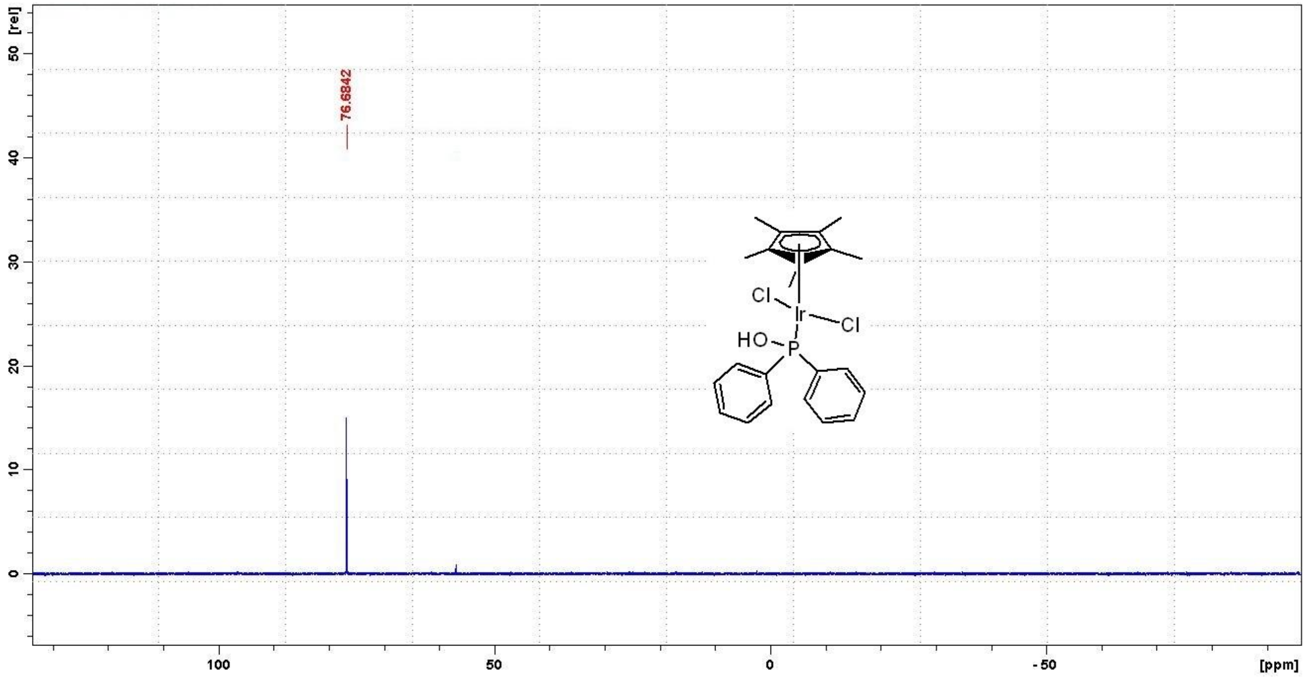

Figure SI-10:^{31}P{^{1}H} NMR spectrum of **5**

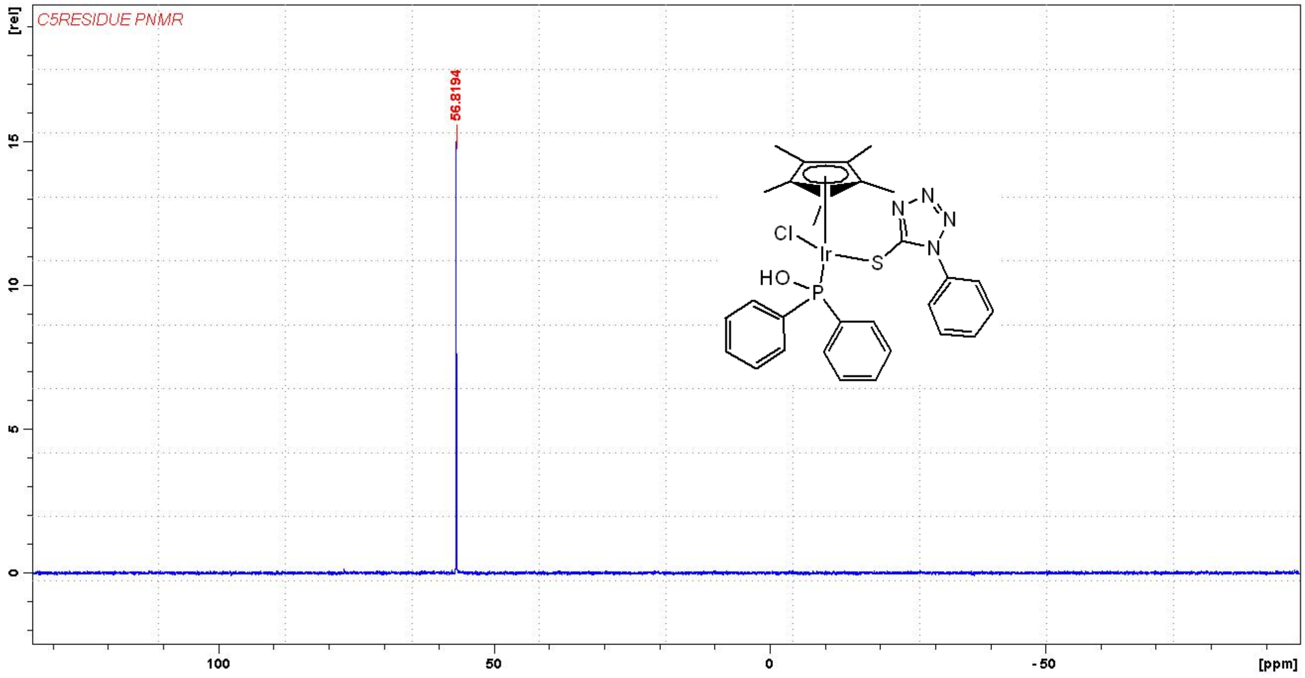

Figure SI-11:^{31}P{^{1}H} NMR spectrum of **8**

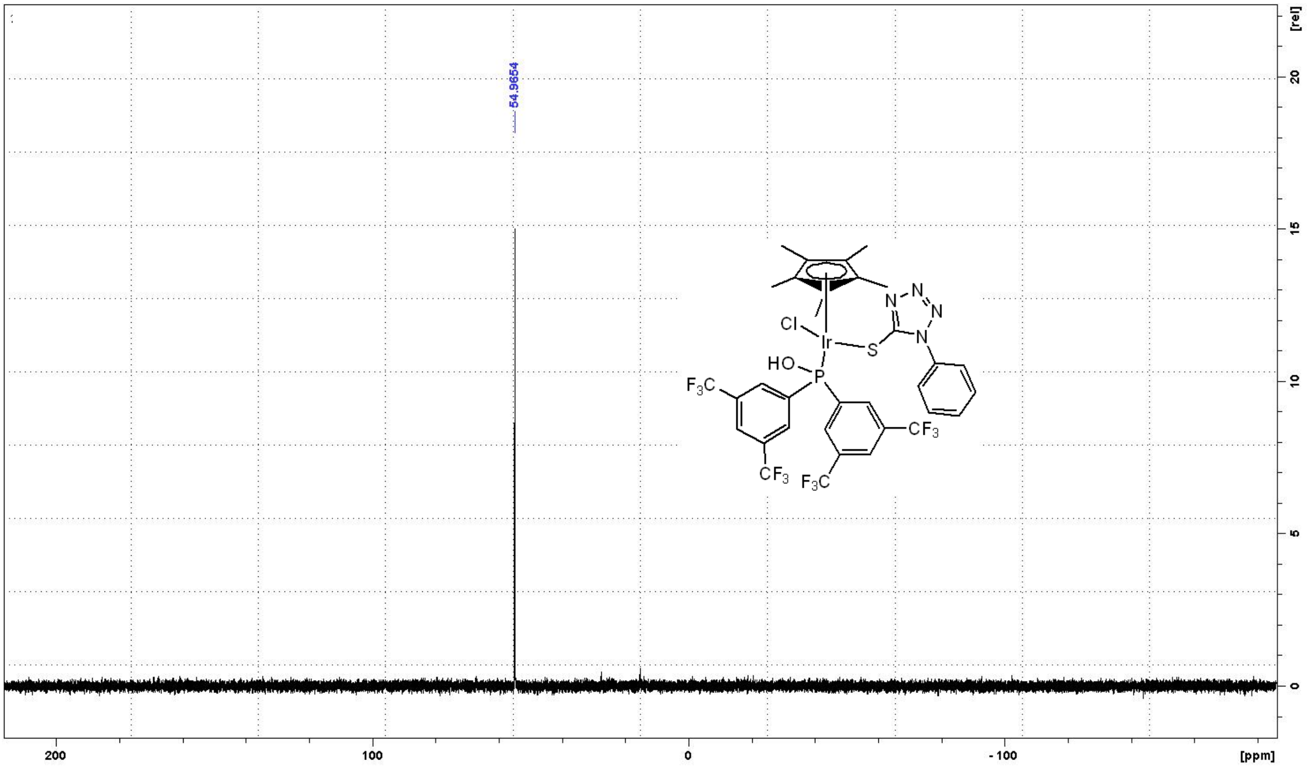

Figure SI-12: ${}^{31}P\{{}^{1}H\}$ NMR spectrum of **9**

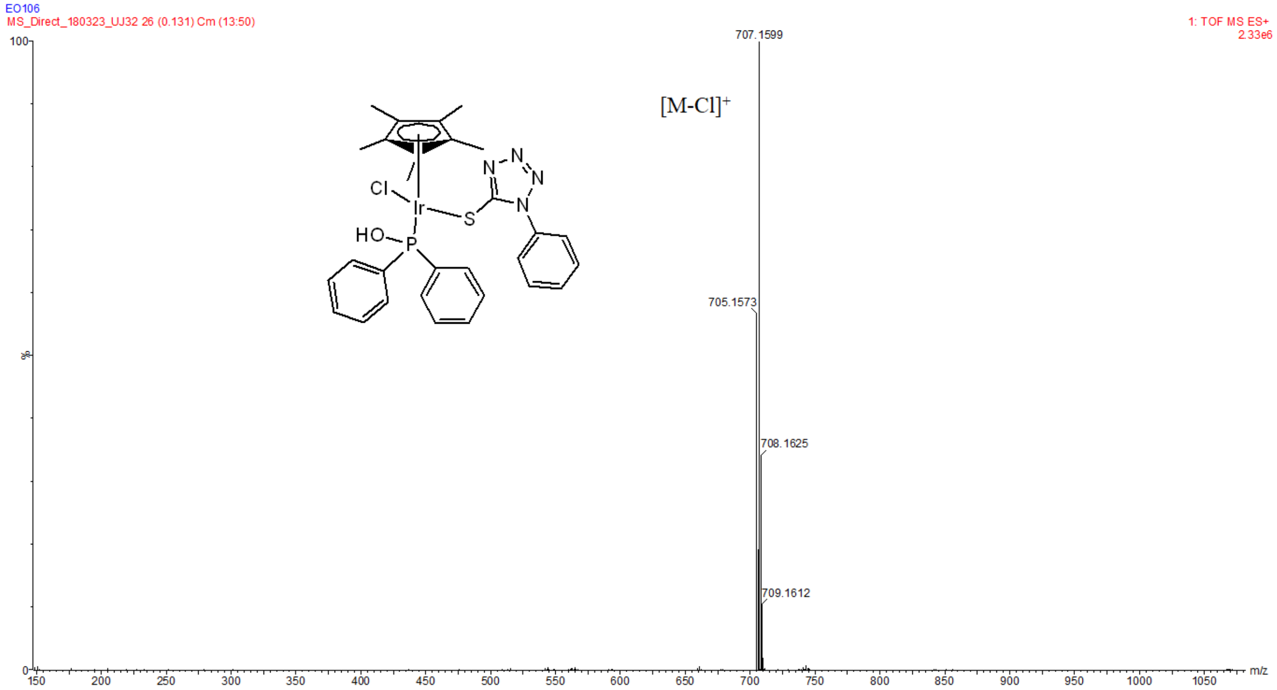

Figure SI-13: Mass spectrum of **8**

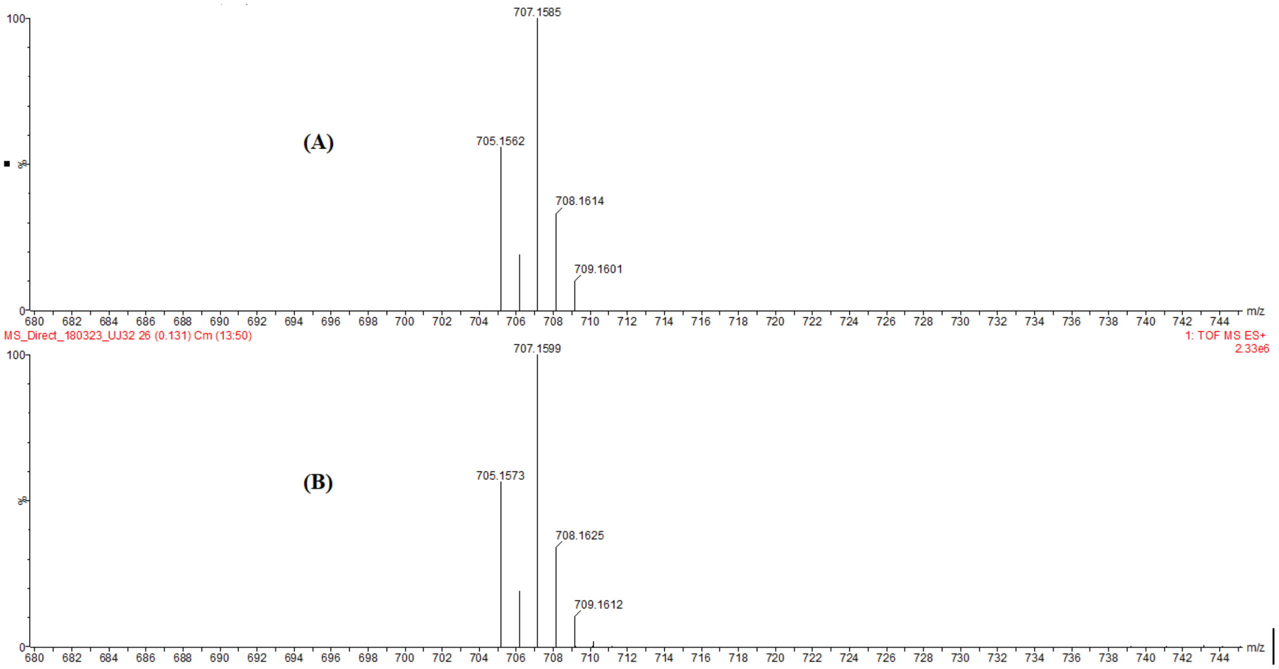

Figure SI-14: Mass spectrum of **8 (A)** predicted **(B)** experimental

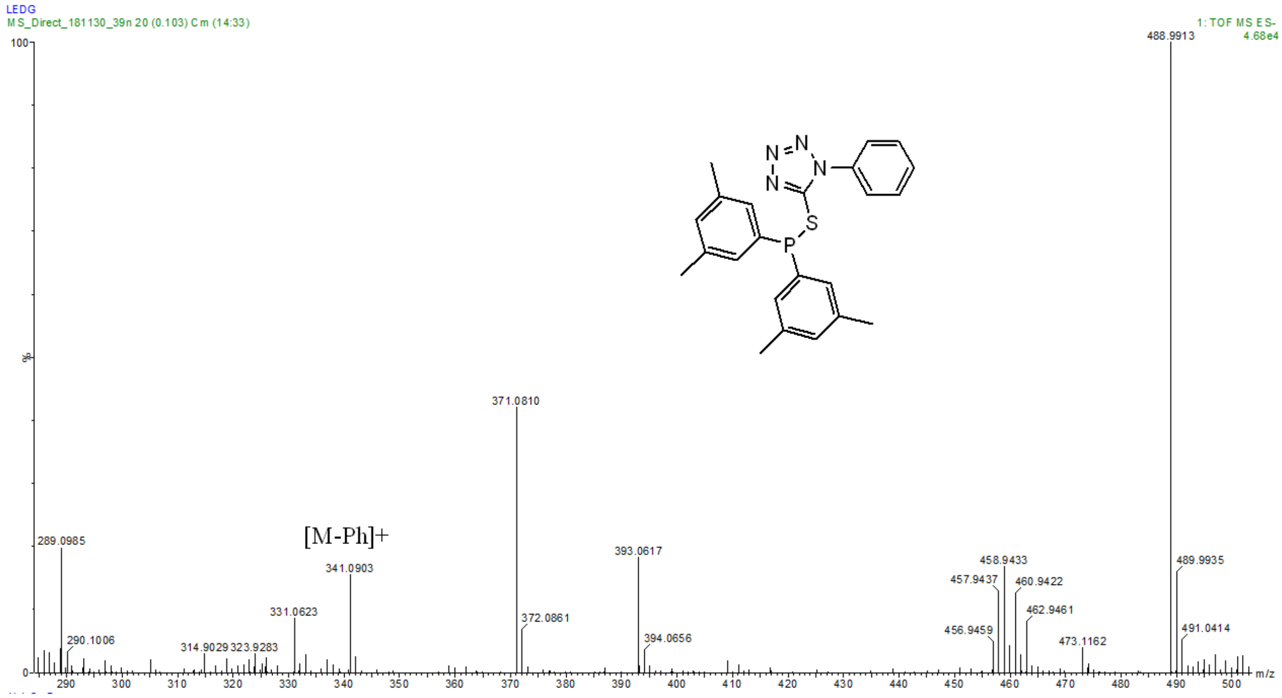

Figure SI-15: Mass spectrum of **1**

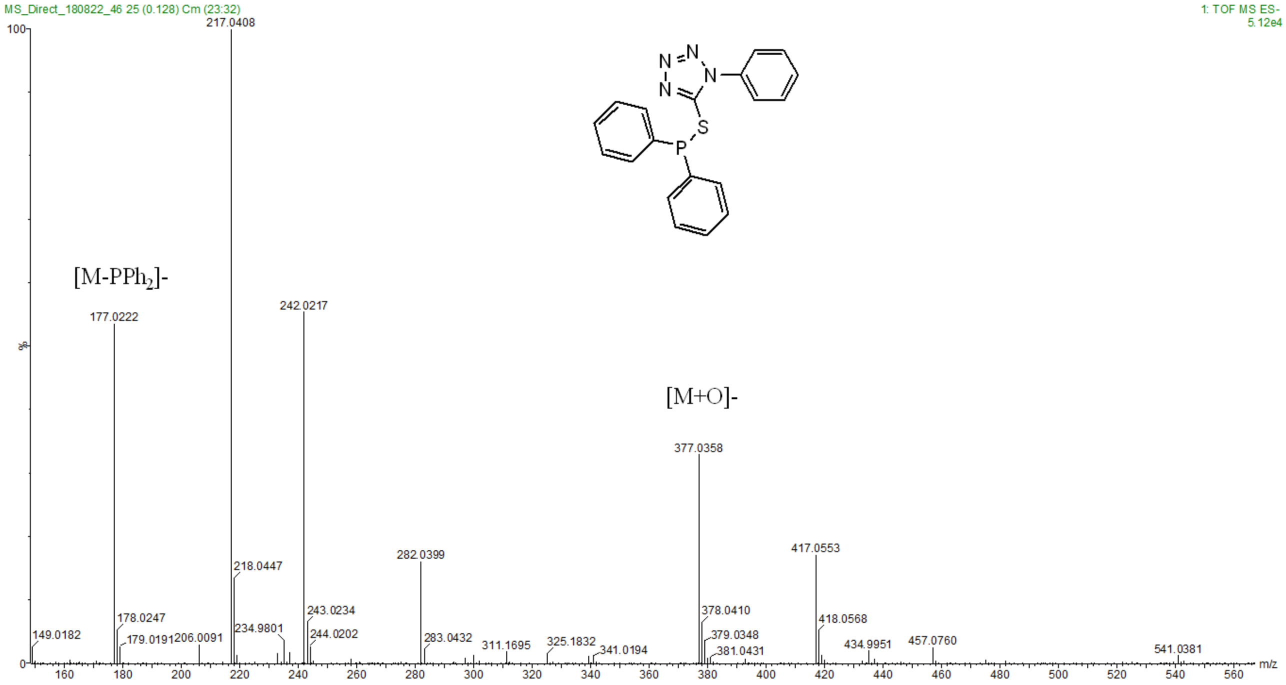

Figure SI-16: Mass spectrum of **2**

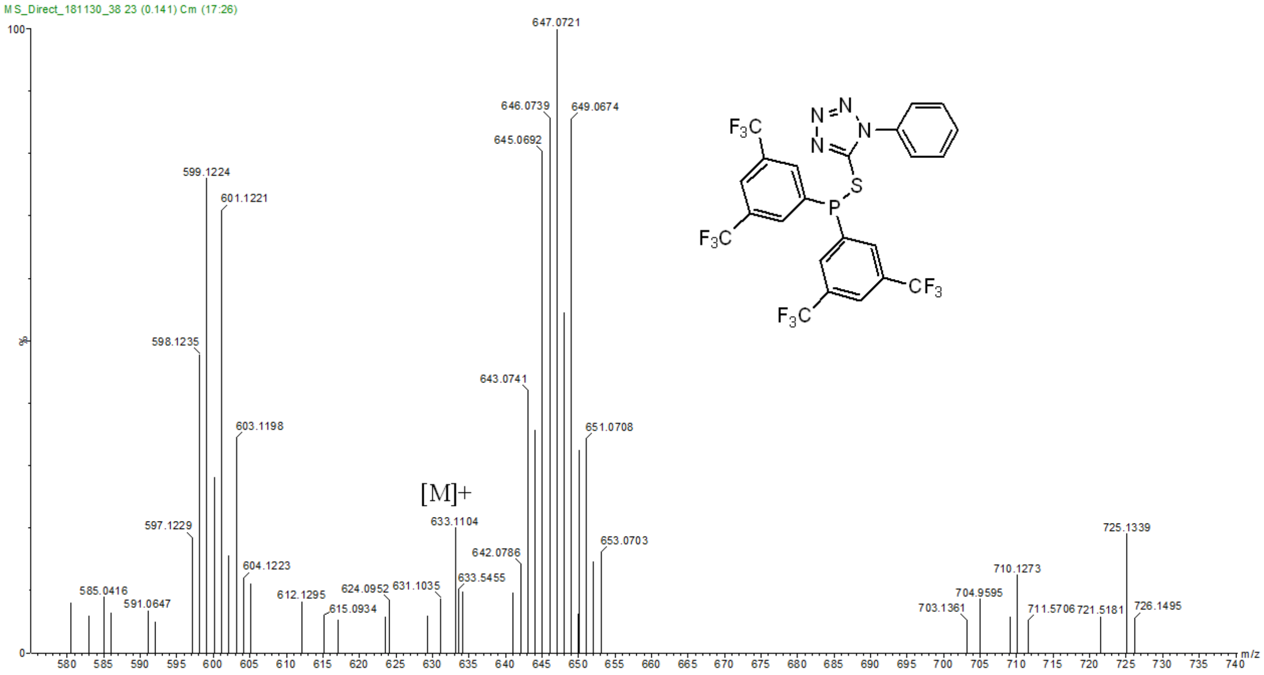

Figure SI-17: Mass spectrum of **3**

Table S1: Selected bond lengths and angles for racemic complex **8** and enantiopure complex **8**

	Racemic Complex 8		Enantiopure Complex 8
**Ir(1)-Cl(1)**	2.469(11)	**Ir(1)-Cl(1)**	2.420(19)
**Ir(1)-S(1)**	2.376(10)	**Ir(1)-S(1)**	2.367(2)
**Ir(1)-P(1)**	2.252(10)	**Ir(1)-P(1)**	2.267(18)
**Ir(2)-Cl(2)**	2.462(10)	**S(1)Ir(1)-Cl(1)**	84.38(7)
**Ir(2)-S(2)**	2.373(10)	**P(1)Ir(1)-Cl(1)**	93.58(7)
**Ir(2)-P(2)**	2.261(10)	**P(1)Ir(1)-S(1)**	91.35(7)
**S(1)Ir(1)-Cl(1)**	85.9(3)		
**P(1)Ir(1)-Cl(1)**	97.3(4)		
**P(1)Ir(1)-S(1)**	91.9(3)		
**P(2)Ir(2)-Cl(2)**	97.5(3)		
**P(2)Ir(2)-S(2)**	91.7(3)		
**S(2)Ir(2)-Cl(2)**	85.8(3)		

Table S2: Crystallographic data for racemic complex **8** and enantiopure complex **8**

Identification code	**Racemic complex 8**	**Enantiopure complex 8.CHCl$_3$**
Empirical formula	$C_{29}H_{35}ClIrN_4O_2PS$	$C_{30}H_{36}Cl_4 IrN_4O_2PS$
Formula weight	762.29	860.04
Temperature/K	100.03	100.01
Crystal system	triclinic	monoclinic
Space group	P-1	P2$_1$/n
a/Å	9.154(5)	9.1975(8)
b/Å	11.247(6)	31.652(3)
c/Å	31.151(15)	11.2380(10)
α/°	89.884(7)	90
β/°	90.021(7)	92.385(2)
γ/°	91.789(5)	90
Volume/Å^3	3205(3)	3268.8(5)

Z	4	19
ρ_{calc}g/cm^3	1.580	1.7508
μ/mm^{-1}	4.395	4.556
F(000)	1512.0	1694.6
Crystal size/mm^3	0.3 × 0.2 × 0.2	0.33 × 0.21 × 0.19
Radiation	MoKα (λ = 0.71073)	Mo Kα (λ = 0.71073)
2Θ range for data collection/°	2.614 to 58.752	3.84 to 56.54
Index ranges	-12 ≤ h ≤ 12, -14 ≤ k ≤ 14, -41 ≤ l ≤ 41	-12 ≤ h ≤ 12, -42 ≤ k ≤ 42, -14 ≤ l ≤ 14
Reflections collected	101594	82558
Independent reflections	15825 [R_{int} = 0.1542, R_{sigma} = 0.1262]	8097 [R_{int} = 0.0767, R_{sigma} = 0.0434]
Data/restraints/parameters	15825/0/332	8097/0/385
Goodness-of-fit on F^2	1.234	1.273
Final R indexes [I>=2σ (I)]	R_1 = 0.2126, wR_2 = 0.4799	R_1 = 0.0541, wR_2 = 0.1692
Final R indexes [all data]	R_1 = 0.2547, wR_2 = 0.5063	R_1 = 0.0721, wR_2 = 0.1797
Largest diff. peak/hole / e Å^{-3}	20.87/-11.62	2.56/-2.14
