# Supplementary material for: Chiral-at-Metal: Iridium(III) Tetrazole Complexes With Proton-Responsive P-OH Groups for CO2 Hydrogenation
Source: Front Chem. 2020 Nov 13;8:591353. doi: 10.3389/fchem.2020.591353 (PMC7692406; doi:10.3389/fchem.2020.591353)

checkCIF/PLATON report

Structure factors have been supplied for datablock(s) c5resc

THIS REPORT IS FOR GUIDANCE ONLY. IF USED AS PART OF A REVIEW PROCEDURE FOR PUBLICATION, IT SHOULD NOT REPLACE THE EXPERTISE OF AN EXPERIENCED CRYSTALLOGRAPHIC REFEREE.

No syntax errors found. CIF dictionary Interpreting this report

Datablock: c5resc

Bond precision: C-C = 0.0462 A Wavelength=0.71073

Cell:	a=9.154(5)	b=11.247(6)	c=31.151(15)
	alpha=89.884(7)	beta=90.021(7)	gamma=91.789(5)
Temperature:	100 K		

	Calculated	Reported
Volume	3206(3)	3205(3)
Space group	P -1	P -1
Hall group	-P 1	-P 1
Moiety formula	C29 H34 Cl Ir N4 O P S, C29 H32 Cl Ir N4 O P S, H2 O, H O [+ so	0.5(C29 H32 Cl Ir N4 O P S), 0.5(C29 H34 Cl Ir N4 O P S), 0.5(H
Sum formula	C58 H69 Cl2 Ir2 N8 O4 P2 S2 [+ solvent]	C29 H35 Cl Ir N4 O2 P S
Mr	1523.61	762.29
Dx,g cm-3	1.578	1.580
Z	2	4
Mu (mm-1)	4.394	4.395
F000	1510.0	1512.0
F000’	1506.28	
h,k,lmax	12,15,43	12,14,41
Nref	17689	15825
Tmin,Tmax	0.362,0.415	0.518,0.746
Tmin’	0.257	

Correction method= # Reported T Limits: Tmin=0.518 Tmax=0.746
AbsCorr = MULTI-SCAN

Data completeness= 0.895 Theta(max)= 29.376

R(reflections)= 0.1752(11098) wR2(reflections)= 0.4514(15825)

S = 1.072 Npar= 722

The following ALERTS were generated. Each ALERT has the format
test-name_ALERT_alert-type_alert-level.
Click on the hyperlinks for more details of the test.

Alert level A

```
SHFSU01_ALERT_2_A  The absolute value of parameter shift to su ratio > 0.20
            Absolute value of the parameter shift to su ratio given   1.029
            Additional refinement cycles may be required.
PLAT080_ALERT_2_A Maximum Shift/Error ............................       1.03 Why ?
PLAT211_ALERT_2_A ADP of Atom N00K      is N.P.D. or (nearly) 2D .     Please Check
PLAT211_ALERT_2_A ADP of Atom N00Z      is N.P.D. or (nearly) 2D .     Please Check
PLAT211_ALERT_2_A ADP of Atom C00D      is N.P.D. or (nearly) 2D .     Please Check
PLAT211_ALERT_2_A ADP of Atom C00E      is N.P.D. or (nearly) 2D .     Please Check
PLAT211_ALERT_2_A ADP of Atom C016      is N.P.D. or (nearly) 2D .     Please Check
PLAT211_ALERT_2_A ADP of Atom C01H      is N.P.D. or (nearly) 2D .     Please Check
PLAT211_ALERT_2_A ADP of Atom C01P      is N.P.D. or (nearly) 2D .     Please Check
PLAT211_ALERT_2_A ADP of Atom C01U      is N.P.D. or (nearly) 2D .     Please Check
PLAT211_ALERT_2_A ADP of Atom C01W      is N.P.D. or (nearly) 2D .     Please Check
PLAT211_ALERT_2_A ADP of Atom C01Z      is N.P.D. or (nearly) 2D .     Please Check
PLAT211_ALERT_2_A ADP of Atom C022      is N.P.D. or (nearly) 2D .     Please Check
PLAT211_ALERT_2_A ADP of Atom C023      is N.P.D. or (nearly) 2D .     Please Check
PLAT211_ALERT_2_A ADP of Atom C00M      is N.P.D. or (nearly) 2D .     Please Check
PLAT211_ALERT_2_A ADP of Atom C012      is N.P.D. or (nearly) 2D .     Please Check
PLAT211_ALERT_2_A ADP of Atom C014      is N.P.D. or (nearly) 2D .     Please Check
PLAT211_ALERT_2_A ADP of Atom C015      is N.P.D. or (nearly) 2D .     Please Check
PLAT211_ALERT_2_A ADP of Atom C01G      is N.P.D. or (nearly) 2D .     Please Check
PLAT211_ALERT_2_A ADP of Atom C01O      is N.P.D. or (nearly) 2D .     Please Check
PLAT211_ALERT_2_A ADP of Atom C01R      is N.P.D. or (nearly) 2D .     Please Check
PLAT211_ALERT_2_A ADP of Atom C01X      is N.P.D. or (nearly) 2D .     Please Check
PLAT211_ALERT_2_A ADP of Atom C021      is N.P.D. or (nearly) 2D .     Please Check
PLAT213_ALERT_2_A Atom C00C           has ADP max/min Ratio .....        7.3 oblate
PLAT213_ALERT_2_A Atom C00V           has ADP max/min Ratio .....        5.7 prolat
PLAT213_ALERT_2_A Atom C01F           has ADP max/min Ratio .....        6.7 prolat
PLAT213_ALERT_2_A Atom C025           has ADP max/min Ratio .....        7.9 prolat
PLAT213_ALERT_2_A Atom N00P           has ADP max/min Ratio .....        9.1 prolat
PLAT213_ALERT_2_A Atom C00R           has ADP max/min Ratio .....       10.1 prolat
PLAT213_ALERT_2_A Atom C017           has ADP max/min Ratio .....        6.3 prolat
PLAT213_ALERT_2_A Atom C018           has ADP max/min Ratio .....        6.3 prolat
PLAT213_ALERT_2_A Atom C01E           has ADP max/min Ratio .....       20.0 prolat
PLAT330_ALERT_2_A Large Average Phenyl C-C Dist C00G     -C018          1.45 Ang.
PLAT417_ALERT_2_A Short Inter D-H..H-D       H8       ..H8              1.55 Ang.
PLAT973_ALERT_2_A Check Calcd Positive Resid. Density on      Ir01       3.64 eA-3
PLAT973_ALERT_2_A Check Calcd Positive Resid. Density on      Ir02       3.30 eA-3
```

Alert level B

```
PLAT082_ALERT_2_B High R1 Value ..................................       0.18 Report
PLAT084_ALERT_3_B High wR2 Value (i.e. > 0.25) ...................       0.45 Report
PLAT097_ALERT_2_B Large Reported Max.  (Positive) Residual Density      12.35 eA-3
PLAT212_ALERT_2_B ADP of Atom O1        is N.P.D. or (nearly) 2D .     Please Check
PLAT213_ALERT_2_B Atom C011           has ADP max/min Ratio .....        4.3 prolat
PLAT213_ALERT_2_B Atom C01B           has ADP max/min Ratio .....        4.1 prolat
PLAT213_ALERT_2_B Atom N01D           has ADP max/min Ratio .....        4.2 prolat
PLAT213_ALERT_2_B Atom C02C           has ADP max/min Ratio .....        4.3 prolat
PLAT234_ALERT_4_B Large Hirshfeld Difference C00N     --C00O            0.26 Ang.
PLAT342_ALERT_3_B Low Bond Precision on  C-C Bonds ...............    0.04625 Ang.
PLAT416_ALERT_2_B Short Intra D-H..H-D       H00J     ..H00T            1.03 Ang.
PLAT416_ALERT_2_B Short Intra D-H..H-D       H00H     ..H00I            1.32 Ang.
PLAT420_ALERT_2_B D-H Without Acceptor       O1       --H1B            Please Check
```

Alert level C

```
ABSTY02_ALERT_1_C  An _exptl_absorpt_correction_type has been given without
            a literature citation. This should be contained in the
            _exptl_absorpt_process_details field.
            Absorption correction given as multi-scan
DIFMX02_ALERT_1_C  The maximum difference density is > 0.1*ZMAX*0.75
            The relevant atom site should be identified.
RINTA01_ALERT_3_C  The value of Rint is greater than 0.12
            Rint given   0.154
PLAT020_ALERT_3_C The Value of Rint is Greater Than 0.12 .........      0.154 Report
PLAT094_ALERT_2_C Ratio of Maximum / Minimum Residual Density ....       2.23 Report
PLAT155_ALERT_4_C The Triclinic Unitcell is NOT Reduced ..........     Please Do !
PLAT213_ALERT_2_C Atom C00O            has ADP max/min Ratio .....        3.9 prolat
PLAT213_ALERT_2_C Atom C01Y            has ADP max/min Ratio .....        3.8 prolat
PLAT213_ALERT_2_C Atom C00Y            has ADP max/min Ratio .....        3.3 prolat
PLAT213_ALERT_2_C Atom C010            has ADP max/min Ratio .....        3.5 oblate
PLAT213_ALERT_2_C Atom C01J            has ADP max/min Ratio .....        3.3 prolat
PLAT220_ALERT_2_C Non-Solvent  Resd 1  C   Ueq(max)/Ueq(min) Range        3.3 Ratio
PLAT220_ALERT_2_C Non-Solvent  Resd 2  C   Ueq(max)/Ueq(min) Range        3.2 Ratio
PLAT234_ALERT_4_C Large Hirshfeld Difference S006     --C01B             0.17 Ang.
PLAT234_ALERT_4_C Large Hirshfeld Difference C00U     --C00W             0.22 Ang.
PLAT234_ALERT_4_C Large Hirshfeld Difference C013     --C01U             0.24 Ang.
PLAT241_ALERT_2_C High   'MainMol' Ueq as Compared to Neighbors of       C016 Check
PLAT241_ALERT_2_C High   'MainMol' Ueq as Compared to Neighbors of       C01H Check
PLAT241_ALERT_2_C High   'MainMol' Ueq as Compared to Neighbors of       C022 Check
PLAT241_ALERT_2_C High   'MainMol' Ueq as Compared to Neighbors of       C00X Check
PLAT241_ALERT_2_C High   'MainMol' Ueq as Compared to Neighbors of       C019 Check
PLAT241_ALERT_2_C High   'MainMol' Ueq as Compared to Neighbors of       C01E Check
PLAT241_ALERT_2_C High   'MainMol' Ueq as Compared to Neighbors of       C027 Check
PLAT242_ALERT_2_C Low    'MainMol' Ueq as Compared to Neighbors of       C00V Check
PLAT242_ALERT_2_C Low    'MainMol' Ueq as Compared to Neighbors of       C01P Check
PLAT242_ALERT_2_C Low    'MainMol' Ueq as Compared to Neighbors of       C01Z Check
PLAT242_ALERT_2_C Low    'MainMol' Ueq as Compared to Neighbors of       C028 Check
PLAT250_ALERT_2_C Large U3/U1 Ratio for Average U(i,j) Tensor ....        3.4 Note
PLAT250_ALERT_2_C Large U3/U1 Ratio for Average U(i,j) Tensor ....        2.8 Note
PLAT330_ALERT_2_C Large Average Phenyl C-C Dist C00L     -C013           1.41 Ang.
PLAT332_ALERT_2_C Large Phenyl C-C Range       C00Y     -C01T            0.22 Ang.
PLAT368_ALERT_2_C Short  C(sp2)-C(sp2) Bond  C016     - C01Z     .       1.21 Ang.
PLAT369_ALERT_2_C Long   C(sp2)-C(sp2) Bond  C00E     - C01M     .       1.53 Ang.
PLAT369_ALERT_2_C Long   C(sp2)-C(sp2) Bond  C016     - C01P     .       1.55 Ang.
PLAT374_ALERT_2_C Long   N - N         Bond  N00T     - N00Z     .       1.51 Ang.
PLAT420_ALERT_2_C D-H Without Acceptor       N00S     --H00S           Please Check
PLAT420_ALERT_2_C D-H Without Acceptor       N00Z     --H00Z           Please Check
PLAT906_ALERT_3_C Large K Value in the Analysis of Variance ......      6.354 Check
PLAT906_ALERT_3_C Large K Value in the Analysis of Variance ......      4.056 Check
PLAT906_ALERT_3_C Large K Value in the Analysis of Variance ......      2.669 Check
PLAT911_ALERT_3_C Missing FCF Refl Between Thmin & STh/L=    0.600         17 Report
PLAT918_ALERT_3_C Reflection(s) with I(obs) much Smaller I(calc) .          3 Check
PLAT977_ALERT_2_C Check Negative Difference Density on H00A             -0.77 eA-3
PLAT977_ALERT_2_C Check Negative Difference Density on H00B             -0.32 eA-3
PLAT977_ALERT_2_C Check Negative Difference Density on H00C             -1.02 eA-3
PLAT977_ALERT_2_C Check Negative Difference Density on H00H             -0.88 eA-3
PLAT977_ALERT_2_C Check Negative Difference Density on H00S             -1.13 eA-3
PLAT977_ALERT_2_C Check Negative Difference Density on H00Z             -0.71 eA-3
PLAT977_ALERT_2_C Check Negative Difference Density on H010             -0.37 eA-3
PLAT977_ALERT_2_C Check Negative Difference Density on H01I             -0.32 eA-3
PLAT977_ALERT_2_C Check Negative Difference Density on H01J             -1.12 eA-3
PLAT977_ALERT_2_C Check Negative Difference Density on H01H             -0.40 eA-3
PLAT977_ALERT_2_C Check Negative Difference Density on H01R             -0.80 eA-3
PLAT977_ALERT_2_C Check Negative Difference Density on H01X             -0.49 eA-3
PLAT977_ALERT_2_C Check Negative Difference Density on H1AA             -0.71 eA-3
```

```
PLAT977_ALERT_2_C Check Negative Difference Density on H01U            -0.48 eA-3
PLAT977_ALERT_2_C Check Negative Difference Density on H01V            -1.32 eA-3
PLAT977_ALERT_2_C Check Negative Difference Density on H02H            -0.32 eA-3
PLAT977_ALERT_2_C Check Negative Difference Density on H02B            -0.58 eA-3
PLAT977_ALERT_2_C Check Negative Difference Density on H02E            -0.97 eA-3
PLAT977_ALERT_2_C Check Negative Difference Density on H026            -0.57 eA-3
PLAT978_ALERT_2_C Number C-C Bonds with Positive Residual Density.          0 Info
```

Alert level G

```
FORMU01_ALERT_1_G  There is a discrepancy between the atom counts in the
            _chemical_formula_sum and _chemical_formula_moiety. This is
            usually due to the moiety formula being in the wrong format.
            Atom count from _chemical_formula_sum:   C29 H35 Cl1 Ir1 N4 O2 P1 S1
            Atom count from _chemical_formula_moiety:C29 H34.5 Cl1 Ir1 N4 O1.5 P
FORMU01_ALERT_2_G  There is a discrepancy between the atom counts in the
            _chemical_formula_sum and the formula from the _atom_site* data.
            Atom count from _chemical_formula_sum:C29 H35 Cl1 Ir1 N4 O2 P1 S1
            Atom count from the _atom_site data:  C29 H34.5 Cl1 Ir1 N4 O2 P1 S1
CELLZ01_ALERT_1_G Difference between formula and atom_site contents detected.
CELLZ01_ALERT_1_G WARNING: H atoms missing from atom site list. Is this intentional?
           From the CIF: _cell_formula_units_Z    4
           From the CIF: _chemical_formula_sum  C29 H35 Cl Ir N4 O2 P S
           TEST: Compare cell contents of formula and atom_site data
```

atom	Z*formula	cif sites	diff
C	116.00	116.00	0.00
H	140.00	138.00	2.00
Cl	4.00	4.00	0.00
Ir	4.00	4.00	0.00
N	16.00	16.00	0.00
O	8.00	8.00	0.00
P	4.00	4.00	0.00
S	4.00	4.00	0.00

```
PLAT007_ALERT_5_G Number of Unrefined Donor-H Atoms .............          9 Report
PLAT012_ALERT_1_G No       _shelx_res_checksum Found in CIF ......     Please Check
PLAT041_ALERT_1_G Calc. and Reported SumFormula    Strings  Differ     Please Check
PLAT042_ALERT_1_G Calc. and Reported MoietyFormula Strings  Differ     Please Check
PLAT045_ALERT_1_G Calculated and Reported Z Differ by a Factor ...       0.50 Check
PLAT068_ALERT_1_G Reported F000 Differs from Calcd (or Missing)...     Please Check
PLAT072_ALERT_2_G SHELXL First  Parameter in WGHT  Unusually Large       0.11 Report
PLAT083_ALERT_2_G SHELXL Second Parameter in WGHT  Unusually Large     656.75 Why ?
PLAT112_ALERT_2_G ADDSYM Detects New (Pseudo) Symm. Elem        n          97 %Fit
PLAT113_ALERT_2_G ADDSYM Suggests Possible Pseudo/New  Space Group      P21/n Check
PLAT432_ALERT_2_G Short Inter X...Y Contact  C01X     ..C022             3.13 Ang.
PLAT605_ALERT_4_G Largest Solvent Accessible VOID in the Structure        231 A**3
PLAT720_ALERT_4_G Number of Unusual/Non-Standard Labels ..........        141 Note
PLAT790_ALERT_4_G Centre of Gravity not Within Unit Cell: Resd.  #          3 Note
              H2 O
PLAT790_ALERT_4_G Centre of Gravity not Within Unit Cell: Resd.  #          4 Note
              H O
PLAT793_ALERT_4_G Model has Chirality at P008        (Centro SPGR)          S Verify
PLAT868_ALERT_4_G ALERTS Due to the Use of _smtbx_masks Suppressed          ! Info
PLAT910_ALERT_3_G Missing # of FCF Reflection(s) Below Theta(Min).          1 Note
PLAT912_ALERT_4_G Missing # of FCF Reflections Above STh/L=  0.600       1702 Note
PLAT952_ALERT_5_G Calculated (ThMax) and CIF-Reported Lmax Differ           2 Units
PLAT958_ALERT_1_G Calculated (ThMax) and Actual (FCF) Lmax Differ           2 Units
```

---

36 **ALERT level A** = Most likely a serious problem - resolve or explain
13 **ALERT level B** = A potentially serious problem, consider carefully
62 **ALERT level C** = Check. Ensure it is not caused by an omission or oversight

```
 25 ALERT level G = General information/check it is not something unexpected

 11 ALERT type 1 CIF construction/syntax error, inconsistent or missing data
101 ALERT type 2 Indicator that the structure model may be wrong or deficient
 10 ALERT type 3 Indicator that the structure quality may be low
 12 ALERT type 4 Improvement, methodology, query or suggestion
  2 ALERT type 5 Informative message, check
```

It is advisable to attempt to resolve as many as possible of the alerts in all categories. Often the minor alerts point to easily fixed oversights, errors and omissions in your CIF or refinement strategy, so attention to these fine details can be worthwhile. In order to resolve some of the more serious problems it may be necessary to carry out additional measurements or structure refinements. However, the purpose of your study may justify the reported deviations and the more serious of these should normally be commented upon in the discussion or experimental section of a paper or in the "special_details" fields of the CIF. checkCIF was carefully designed to identify outliers and unusual parameters, but every test has its limitations and alerts that are not important in a particular case may appear. Conversely, the absence of alerts does not guarantee there are no aspects of the results needing attention. It is up to the individual to critically assess their own results and, if necessary, seek expert advice.

Publication of your CIF in IUCr journals

A basic structural check has been run on your CIF. These basic checks will be run on all CIFs submitted for publication in IUCr journals (*Acta Crystallographica*, *Journal of Applied Crystallography*, *Journal of Synchrotron Radiation*); however, if you intend to submit to *Acta Crystallographica Section C* or *E* or *IUCrData*, you should make sure that full publication checks are run on the final version of your CIF prior to submission.

Publication of your CIF in other journals

Please refer to the *Notes for Authors* of the relevant journal for any special instructions relating to CIF submission.

PLATON version of 23/04/2018; check.def file version of 23/04/2018

Datablock c5resc - ellipsoid plot

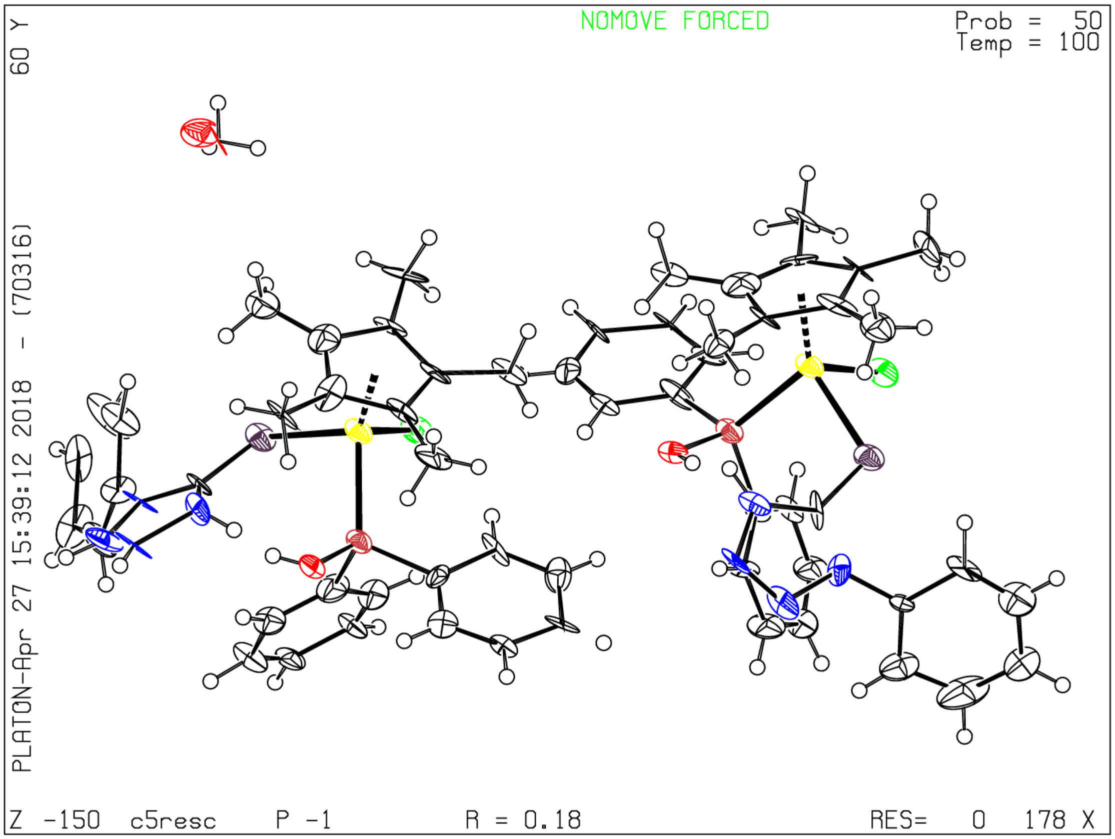

Supplement: Supplementary file 2 [file Data_Sheet_1.PDF]
