# Supplementary material for: Chiral-at-Metal: Iridium(III) Tetrazole Complexes With Proton-Responsive P-OH Groups for CO2 Hydrogenation
Source: Front Chem. 2020 Nov 13;8:591353. doi: 10.3389/fchem.2020.591353 (PMC7692406; doi:10.3389/fchem.2020.591353)

checkCIF/PLATON report

Structure factors have been supplied for datablock(s) mo_gift_c5r_0m

THIS REPORT IS FOR GUIDANCE ONLY. IF USED AS PART OF A REVIEW PROCEDURE FOR PUBLICATION, IT SHOULD NOT REPLACE THE EXPERTISE OF AN EXPERIENCED CRYSTALLOGRAPHIC REFEREE.

No syntax errors found. CIF dictionary Interpreting this report

Datablock: mo_gift_c5r_0m

```
Bond precision:  C-C = 0.0123 A          Wavelength=0.71073

Cell:            a=9.1975(8)     b=31.652(3)        c=11.238(1)
                 alpha=90        beta=92.385(2)     gamma=90
Temperature:     100 K
```

	Calculated	Reported
Volume	3268.8(5)	3268.8(5)
Space group	P 21/n	P 1 21/n 1
Hall group	-P 2yn	-P 2ybc (x-
Moiety formula	C29 H31 Cl Ir N4 O P S, C H Cl3	0.21(C29 H31 Cl Ir N4 O P S), 0.21(C H Cl3)
Sum formula	C30 H32 Cl4 Ir N4 O P S	C6.316 H6.737 Cl0.842 Ir0.211 N0.842 O0.211 P0.211 S0.211
Mr	861.65	181.41
Dx,g cm-3	1.751	1.751
Z	4	19
Mu (mm-1)	4.556	4.556
F000	1696.0	1694.6
F000’	1694.04	
h,k,lmax	12,42,14	12,42,14
Nref	8102	8097
Tmin,Tmax	0.348,0.402	0.221,0.734
Tmin’	0.245	

```
Correction method= # Reported T Limits: Tmin=0.221 Tmax=0.734
AbsCorr = MULTI-SCAN

Data completeness= 0.999          Theta(max)= 28.270

R(reflections)= 0.0541( 6431)     wR2(reflections)= 0.1797( 8097)

S = 1.273                   Npar= 385
```

The following ALERTS were generated. Each ALERT has the format
test-name_ALERT_alert-type_alert-level.
Click on the hyperlinks for more details of the test.

Alert level B

```
PLAT934_ALERT_3_B Number of (Iobs-Icalc)/Sigma(W) > 10 Outliers ..          3 Check
```

Alert level C

```
ABSTY02_ALERT_1_C  An _exptl_absorpt_correction_type has been given without
            a literature citation. This should be contained in the
            _exptl_absorpt_process_details field.
            Absorption correction given as multi-scan
PLAT041_ALERT_1_C Calc. and Reported SumFormula    Strings  Differ     Please Check
PLAT126_ALERT_1_C Error in or Uninterpretable Hall Symbol  .......  -P 2YBC   (X-Z,Y
PLAT244_ALERT_4_C Low    Solvent Ueq as Compared to Neighbors of       C016 Check
PLAT260_ALERT_2_C Large Average Ueq of Residue Including       Cl1      0.199 Check
PLAT342_ALERT_3_C Low Bond Precision on  C-C Bonds ...............    0.01232 Ang.
PLAT906_ALERT_3_C Large K Value in the Analysis of Variance ......      2.811 Check
PLAT971_ALERT_2_C Check Calcd Resid. Dens.  0.23A   From Cl2            1.91 eA-3
PLAT971_ALERT_2_C Check Calcd Resid. Dens.  0.11A   From Cl1            1.56 eA-3
PLAT972_ALERT_2_C Check Calcd Resid. Dens.  1.46A   From C014          -2.06 eA-3
PLAT972_ALERT_2_C Check Calcd Resid. Dens.  0.62A   From Cl1           -1.77 eA-3
PLAT972_ALERT_2_C Check Calcd Resid. Dens.  0.59A   From Cl00          -1.74 eA-3
PLAT972_ALERT_2_C Check Calcd Resid. Dens.  0.81A   From Ir01          -1.66 eA-3
PLAT972_ALERT_2_C Check Calcd Resid. Dens.  0.70A   From Cl00          -1.58 eA-3
PLAT972_ALERT_2_C Check Calcd Resid. Dens.  1.12A   From Cl2           -1.55 eA-3
PLAT975_ALERT_2_C Check Calcd Resid. Dens.  1.08A   From O005           0.60 eA-3
PLAT976_ALERT_2_C Check Calcd Resid. Dens.  0.75A   From O005          -0.65 eA-3
PLAT977_ALERT_2_C Check Negative Difference Density on H005            -0.65 eA-3
```

Alert level G

```
FORMU01_ALERT_1_G  There is a discrepancy between the atom counts in the
            _chemical_formula_sum and _chemical_formula_moiety. This is
            usually due to the moiety formula being in the wrong format.
            Atom count from _chemical_formula_sum:   C6.316 H6.737 Cl0.842 Ir0.211
            Atom count from _chemical_formula_moiety:C6.3 H6.72 Cl0.84 Ir0.21 N0.8
PLAT042_ALERT_1_G Calc. and Reported MoietyFormula Strings  Differ     Please Check
PLAT045_ALERT_1_G Calculated and Reported Z Differ by a Factor ...       0.21 Check
PLAT068_ALERT_1_G Reported F000 Differs from Calcd (or Missing)...     Please Check
PLAT073_ALERT_1_G H-atoms ref, but _hydrogen_treatment Reported as     constr Check
PLAT434_ALERT_2_G Short Inter HL..HL Contact Cl2      ..Cl2             2.67 Ang.
                                                 2-x,1-y,-z  =      3_765 Check
PLAT720_ALERT_4_G Number of Unusual/Non-Standard Labels ..........         72 Note
PLAT793_ALERT_4_G Model has Chirality at P02        (Centro SPGR)          R Verify
PLAT910_ALERT_3_G Missing # of FCF Reflection(s) Below Theta(Min).          1 Note
PLAT912_ALERT_4_G Missing # of FCF Reflections Above STh/L=  0.600          5 Note
PLAT960_ALERT_3_G Number of Intensities with I  <   - 2*sig(I) ...          8 Check
PLAT978_ALERT_2_G Number C-C Bonds with Positive Residual Density.          1 Info
PLAT982_ALERT_1_G The Ir-f'=   -1.3039 Deviates from  IT-value =      -1.4442 Check
PLAT982_ALERT_1_G The  P-f'=    0.1043 Deviates from  IT-value =       0.1023 Check
PLAT983_ALERT_1_G The Cl-f"=    0.1603 Deviates from  IT-Value =       0.1585 Check
PLAT983_ALERT_1_G The Ir-f"=    7.9811 Deviates from  IT-Value =       7.9887 Check
PLAT983_ALERT_1_G The  P-f"=    0.0967 Deviates from  IT-Value =       0.0942 Check
PLAT983_ALERT_1_G The  S-f"=    0.1244 Deviates from  IT-Value =       0.1234 Check
```

0 **ALERT level A** = Most likely a serious problem - resolve or explain

```
 1 ALERT level B = A potentially serious problem, consider carefully
18 ALERT level C = Check. Ensure it is not caused by an omission or oversight
18 ALERT level G = General information/check it is not something unexpected

14 ALERT type 1 CIF construction/syntax error, inconsistent or missing data
14 ALERT type 2 Indicator that the structure model may be wrong or deficient
 5 ALERT type 3 Indicator that the structure quality may be low
 4 ALERT type 4 Improvement, methodology, query or suggestion
 0 ALERT type 5 Informative message, check
```

It is advisable to attempt to resolve as many as possible of the alerts in all categories. Often the minor alerts point to easily fixed oversights, errors and omissions in your CIF or refinement strategy, so attention to these fine details can be worthwhile. In order to resolve some of the more serious problems it may be necessary to carry out additional measurements or structure refinements. However, the purpose of your study may justify the reported deviations and the more serious of these should normally be commented upon in the discussion or experimental section of a paper or in the "special_details" fields of the CIF. checkCIF was carefully designed to identify outliers and unusual parameters, but every test has its limitations and alerts that are not important in a particular case may appear. Conversely, the absence of alerts does not guarantee there are no aspects of the results needing attention. It is up to the individual to critically assess their own results and, if necessary, seek expert advice.

Publication of your CIF in IUCr journals

A basic structural check has been run on your CIF. These basic checks will be run on all CIFs submitted for publication in IUCr journals (*Acta Crystallographica*, *Journal of Applied Crystallography*, *Journal of Synchrotron Radiation*); however, if you intend to submit to *Acta Crystallographica Section C* or *E* or *IUCrData*, you should make sure that full publication checks are run on the final version of your CIF prior to submission.

Publication of your CIF in other journals

Please refer to the *Notes for Authors* of the relevant journal for any special instructions relating to CIF submission.

PLATON version of 22/12/2019; check.def file version of 13/12/2019

Datablock mo_gift_c5r_0m - ellipsoid plot

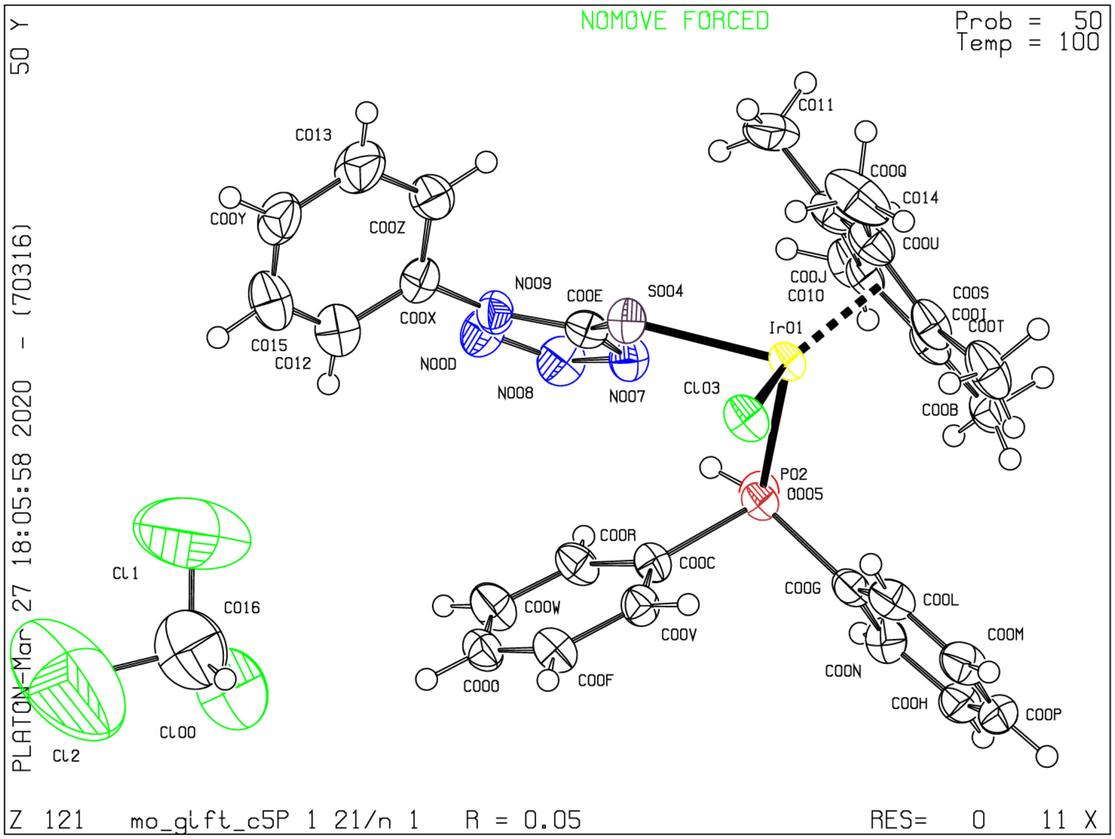

Supplement: Supplementary file 3 [file Data_Sheet_2.PDF]
